# The association between major gastrointestinal cancers and red and processed meat and fish consumption: A systematic review and meta-analysis of the observational studies

**DOI:** 10.1371/journal.pone.0305994

**Published:** 2024-06-26

**Authors:** Jalal Poorolajal, Younes Mohammadi, Marzieh Fattahi-Darghlou, Fatemeh Almasi-Moghadam

**Affiliations:** 1 Department of Epidemiology, School of Public Health, Hamadan University of Medical Sciences, Hamadan, Iran; 2 Modeling of Noncommunicable Diseases Research Center, Hamadan University of Medical Sciences, Hamadan, Iran; 3 Research Center for Health Sciences, Hamadan University of Medical Sciences, Hamadan, Iran; 4 Social Determinants of Health Research Center, Hamadan University of Medical Sciences, Hamadan, Iran; Debre Tabor University, ETHIOPIA

## Abstract

**Background:**

The association between red meat, fish, and processed meat consumption and the risk of developing gastrointestinal (GI) cancers remains inconclusive despite several investigations. Therefore, we conducted a systematic review and meta-analysis of observational studies to update the existing scientific evidence.

**Methods:**

We searched PubMed, Web of Science, and Scopus databases until May 20, 2023. We analyzed observational studies that examined the associations between red and processed meat and fish consumption and GI cancers. We assessed between-study heterogeneity using the χ^2^ and τ^2^ tests, as well as I^2^ statistics. We explored the likelihood of publication bias using Begg’s and Egger’s tests and trim-and-fill analysis. We reported the overall effect sizes as odds ratios (ORs) with a 95% confidence interval (CI) using a random-effects model.

**Results:**

Of the 21,004 studies identified, 95 studies involving 5,794,219 participants were included in the meta-analysis. The consumption of high levels of red meat, as compared to low levels, was found to significantly increase the risk of developing esophageal, pancreatic, liver, colon, rectal, and colorectal cancers. Similarly, the consumption of high levels of processed meat, as compared to low levels, significantly increased the risk of pancreatic, colon, rectal, and colorectal cancers. In contrast, the consumption of high levels of fish, as compared to low levels, significantly reduced the risk of colon, rectal, and colorectal cancers.

**Conclusions:**

This meta-analysis provides updated evidence on the association between red meat, processed meat, and fish consumption and the risk of developing five major types of GI cancers.

## Introduction

Gastrointestinal (GI) cancers are a significant global health concern, accounting for 26% of all cancer cases and 35% of cancer-related deaths [1]. Among the major types of GI cancers, including colorectal, stomach, liver, esophageal, and pancreatic cancers, millions of new cases and deaths are reported worldwide. Colorectal, stomach, liver cancers are ranked third to sixth in terms of incidence [2].

Numerous epidemiological studies have investigated the role of modifiable dietary risk factors in GI cancers in recent years. A healthy diet with high intakes of vegetables, fruits, and whole grains is known to lower the incidence of GI cancers. Conversely, a poor diet with high intakes of fat, processed meat, and alcohol can increase the risk of these types of cancers [3, 4]. Meats make up a significant part of the diet and have been linked to some malignancies, including GI cancers [4–6]. So far, several systematic reviews and meta-analyses have examined the relationship between meat and meat product consumption and GI cancers [4, 5, 7–15]. While several valuable systematic reviews and meta-analyses have investigated the association between meat consumption and GI cancers, these studies have primarily focused on the effect on individual cancers (e.g., colorectal cancer) and lacked a comprehensive analysis across all major GI cancers (stomach, esophagus, etc.). Additionally, existing reviews have employed diverse methodologies and eligibility criteria for different GI cancers, leading to inconsistent conclusions. This lack of a comprehensive analysis across all major GI cancers and the inconsistency in prior findings highlight the need for a systematic review that employs a standardized approach to synthesize the current evidence on the association between red and processed meat and fish consumption with major GI cancers.

The study was conducted to investigate the association between the consumption of red meat, fish, and processed meat and the development of the five major types of GI cancers based on the latest available scientific evidence. This was done because GI cancers pose a major health issue on a global scale, contributing significantly to both the number of cancer instances and fatalities across the world. While numerous epidemiological studies have investigated the role of modifiable dietary risk factors in GI cancers, the evidence for the association between meat products and GI cancers, while generally consistent, is still limited and inconclusive [6]. Therefore, updating the results of previously conducted systematic reviews and meta-analyses with current scientific literature could help make more robust recommendations regarding the consumption of fish and meat products.

## Methods

This study was conducted as a systematic review and meta-analysis; no humans or animals were involved in this research.

### Eligibility criteria (PICOS)

#### Population

The study population consisted of individuals from the general population without any restrictions based on age, gender, race, ethnicity, or nationality.

#### Intervention/Exposure

The exposure of interest was the consumption of red meat, fish, processed meat, or processed fish, classified as tertiles, quartiles, or quintiles.

#### Control

The first tertile, quartile, or quintile was considered the control group.

#### Outcome

The outcome of interest was confirmed cases of major GI cancers through pathological examination, including esophageal, stomach, pancreatic, liver, colon, rectum, or colorectal cancers.

#### Studies

Observational (cross-sectional, case-control, or cohort) studies addressing the association between GI cancers and the consumption of red meat or fish were included, regardless of publication status or language. Studies were required to report the effect size in the form of odds ratio (OR), risk ratio (RR), or hazard ratio (HR) with its corresponding 95% confidence interval (CI).

### Information sources and search

PubMed, Web of Science, and Scopus databases were searched until May 20, 2023, and the reference lists of the included studies were also screened for further eligible studies. The following keywords were used in the search as both “Text Word” and “MeSH term”: (meat or fish) and (esophagus or esophageal or stomach or gastric or pancreas or pancreatic or liver or hepatocellular or gallbladder or intestine or intestinal or bowel or colon or rectum or rectal or colorectum or colorectal) and (cancer or neoplasm or malignancy or tumor).

### Study selection

The search outcomes from various databases were consolidated using EndNote software, and duplicate entries were eliminated. Two authors separately reviewed the titles and abstracts to exclude papers that did not meet the eligibility criteria. The complete texts of potentially relevant studies were obtained for further assessment.

### Data extraction

The extracted data from the relevant studies were entered into an electronic data sheet created using Stata software. The following data were extracted: first author’s name, year of publication, country, language, mean or range of age, gender, type of cancer (esophageal, stomach, pancreatic, liver, colon, rectal, or colorectal), exposure (red meat, fish, processed meat, processed fish), study design (cross-sectional, case-control, cohort), classification of consumption (tertiles, quartiles, quintiles), sample size, analysis of potential confounders (adjusted, unadjusted), and effect size (OR, RR, HR) with their corresponding 95% CI.

### Methodological quality

The methodological quality of the included studies was assessed using the Newcastle-Ottawa Scale (NOS) [16], a well-established tool specifically designed to evaluate the quality of non-randomized studies, such as case-control and cohort studies, commonly used in dietary research. This scale considers three broad aspects of study quality: selection of study groups, comparability of study groups, and assessment of either the exposure or outcome of interest. According to this scale, each study could receive a maximum of nine stars. Studies that obtained seven or more stars were categorized as high-quality, while those that received fewer stars were classified as low-quality.

### Heterogeneity and publication bias

To explore the heterogeneity across studies, the chi-square (χ^2^) test [17] and tau-square (τ^2^) test were used, as well as the I^2^ statistic [18]. Based on the I^2^ value, heterogeneity was classified as low (<50%), moderate (50–74%), or high (≥75%) [19]. The possibility of publication bias was examined using Egger’s test [20] and Begg’s test [21] as well as the "trim and fill" analysis [22].

### Summary measures

The effect measure of choice for this study was the OR. However, some studies reported effect size as RR or HR. According to the GLOBOCAN estimates, the incidence rate of major GI cancers was much less than 0.001 globally [2]. However, some studies reported effect sizes as risk ratios (RR) or hazard ratios (HR). Based on the rarity assumption, when the incidence is rare (<1%), the RR and HR are very similar to the OR [23]. Therefore, we combined all types of effect sizes and reported the overall summary measure as OR using a random-effects model [24]. The data were analyzed using Stata version 14.2 (StataCorp, College Station, TX, USA) at a significance level of 0.05.

## Results

### Description of studies

**Fig 1:**
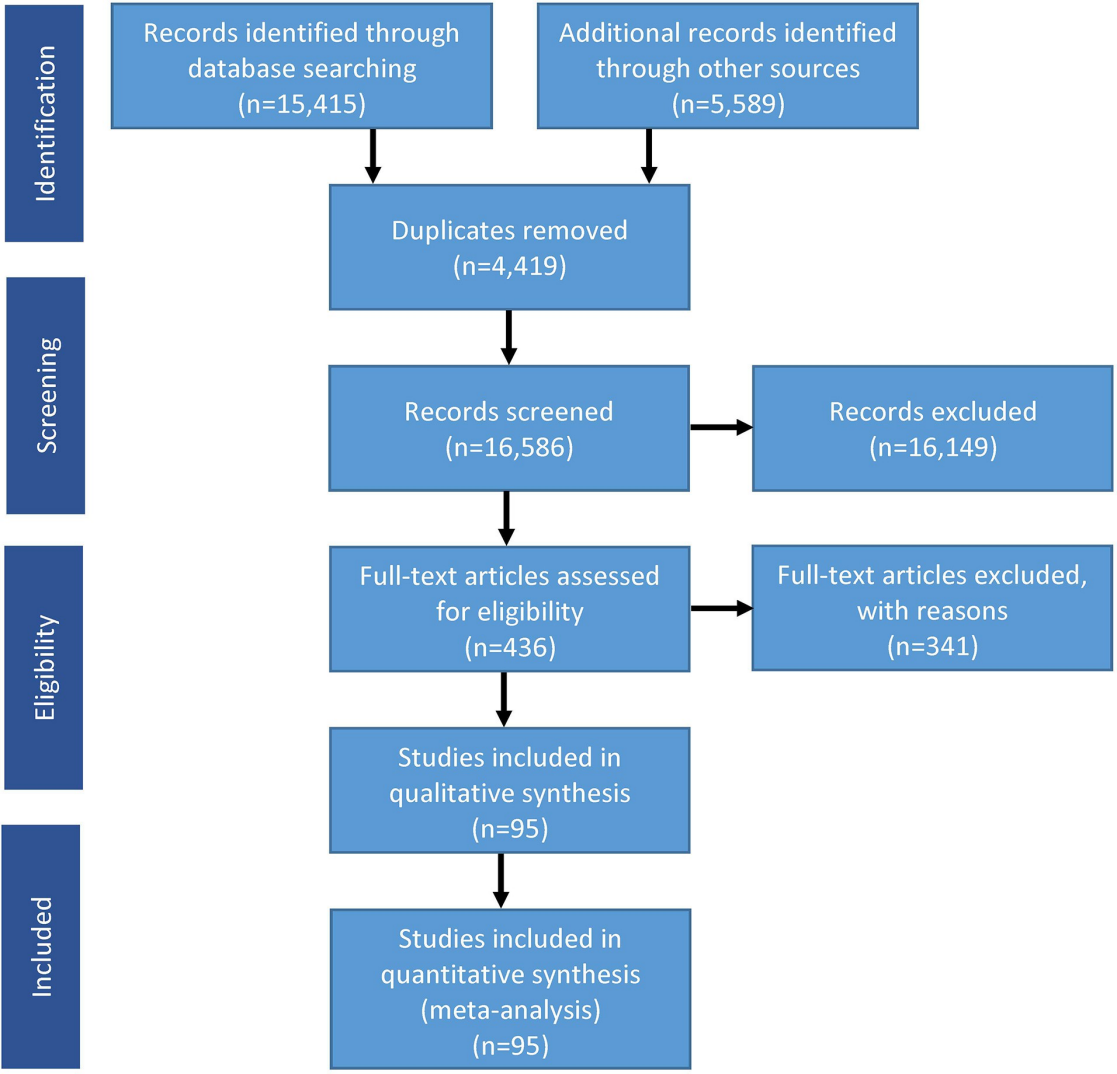
Flow of information through the various phases of the systematic review.

A comprehensive total of 21,004 studies were identified, with 15,415 studies identified through electronic database searches and an additional 5,589 studies found by searching the reference lists of the included studies. After removing duplicate studies and excluding those that did not meet the eligibility criteria, a total of 95 studies involving 5,794,219 participants (S1 Table) were included in the meta-analysis (Fig 1).

### Synthesis of results

The associations between gastrointestinal cancers and red and processed meat and fish consumption are summarized in Table 1. However, for more detailed information on the included studies, Forrest plots are presented in S1 Fig.

**Table 1:** Results of the meta-analysis of the association between gastrointestinal (GI) cancers and consumption of red meat, fish, and processed meat classified as tertiles, quartiles, and quintiles. Protective factors are shown in green color (dark green cells: significant; light green cells: non-significant), and risk factors are shown in red color (dark red cells: significant; light red cells: non-significant).

Cancer	Red meat			Fish			Processed meat		
	No. of study	OR (95% CI)	I^2^	No. of study	OR (95% CI)	I^2^	No. of study	OR (95% CI)	I^2^
**Esophagus**									
1^st^ tertile		Ref.			Ref.			Ref.	
2^nd^ tertile	5	1.380 (0.954, 1.806)	56.9%	3	1.068 (0.790, 1.347)	26.5%	3	0.980 (0.729, 1.230)	00.0%
3^rd^ tertile	5	1.464 (0.806, 2.122)	76.7%	3	0.977 (0.457, 1.496)	77.0%	3	0.946 (0.451, 1.441)	64.9%
1^st^ quartile		Ref.			Ref.			Ref.	
2^nd^ quartile	0	No data	-	1	1.020 (0.565, 1.475)	-	0	No data	-
3^rd^ quartile	0	No data	-	1	1.490 (0.665, 2.315)	-	0	No data	-
4^th^ quartile	0	No data	-	1	1.420 (0.235, 2.605)	-	0	No data	-
1^st^ quintile		Ref.			Ref.			Ref.	
2^nd^ quintile	2	1.318 (0.960, 1.676)	37.4%	2	0.942 (0.733, 1.151)	00.0%	2	0.947 (0.761, 1.133)	00.0%
3^rd^ quintile	2	1.143 (0.893, 1.392)	5.6%	2	0.902 (0.696, 1.108)	00.0%	2	0.951 (0.766, 1.137)	00.0%
4^th^ quintile	2	1.245 (0.986, 1.504)	00.0%	2	0.851 (0.646, 1.056)	00.0%	2	0.973 (0.785, 1.161)	00.0%
5^th^ quintile	2	1.320 (1.039, 1.601)	00.0%	2	0.814 (0.613, 1.014)	00.0%	2	1.032 (0.836, 1.228)	00.0%
**Overall**	**7**	**1.282 (1.128, 1.437)**	**47.5%**	**6**	**0.935 (0.841, 1.030)**	**16.6%**	**5**	**0.961 (0.878, 1.045)**	**00.0%**
**Stomach**									
1^st^ tertile		Ref.			Ref.			Ref.	
2^nd^ tertile	7	1.066 (0.909, 1.224)	21.0%	6	0.887 (0.491, 1.283)	83.8%	7	1.117 (0.987, 1.247)	00.0%
3^rd^ tertile	7	1.243 (1.048, 1.438)	00.0%	6	0.945 (0.667, 1.222)	53.6%	7	1.342 (1.105, 1.579)	20.7%
1^st^ quartile		Ref.			Ref.			Ref.	
2^nd^ quartile	3	0.980 (0.866, 1.093)	5.2%	1	0.710 (0.370, 1.050)	-	2	1.081 (0.906, 1.256)	00.0%
3^rd^ quartile	3	1.063 (0.899, 1.227)	00.0%	1	2.110 (1.010, 3.210)	-	2	1.224 (0.970, 1.479)	00.0%
4^th^ quartile	3	1.136 (0.757, 1.516)	34.4%	1	1.350 (0.245, 2.455)	-	2	1.100 (0.075, 2.276)	92.6%
1^st^ quintile		Ref.			Ref.			Ref.	
2^nd^ quintile	2	0.954 (0.789, 1.119)	00.0%	2	1.057 (0.841, 1.272)	00.0%	2	0.896 (0.742, 1.050)	00.0%
3^rd^ quintile	2	0.853 (0.700, 1.006)	00.0%	2	0.869 (0.678, 1.060)	00.0%	2	0.887 (0.674, 1.100)	47.6%
4^th^ quintile	2	1.000 (0.781, 1.219)	32.7%	2	1.132 (0.896, 1.369)	00.0%	2	0.942 (0.779, 1.104)	00.0%
5^th^ quintile	2	0.925 (0.731, 1.118)	20.6%	2	1.045 (0.819, 1.271)	00.0%	2	0.957 (0.793, 1.122)	00.0%
**Overall**	**12**	**1.019 (0.959, 1.079)**	**17.4%**	**9**	**0.964 (0.832, 1.095)**	**62.8%**	**11**	**1.066 (0.981, 1.151)**	**45.4%**
**Pancreas**									
1^st^ tertile		Ref.			Ref.			Ref.	
2^nd^ tertile	-	No data	-	3	0.943 (0.554, 1.333)	74.9%	0	No data	-
3^rd^ tertile	-	No data	-	3	0.873 (0.659, 1.087)	21.0%	0	No data	-
1^st^ quartile		Ref.			Ref.			Ref.	
2^nd^ quartile	5	1.142 (0.950, 1.335)	28.9%	4	0.965 (0.718, 1.213)	9.1%	1	1.100 (0.800, 1.400)	-
3^rd^ quartile	5	1.048 (0.802, 1.294)	73.0%	4	1.075 (0.842, 1.309)	00.0%	1	1.300 (0.900, 1.700)	-
4^th^ quartile	5	1.017 (0.611, 1.422)	00.0%	4	1.036 (0.797, 1.275)	00.0%	1	1.400 (0.950, 1.850)	-
1^st^ quintile		Ref.			Ref.			Ref.	
2^nd^ quintile	8	1.056 (0.964, 1.148)	00.0%	2	0.960 (0.744, 1.176)	70.7%	4	1.205 (0.993, 1.417)	58.6%
3^rd^ quintile	8	1.104 (1.009, 1.200)	00.0%	2	0.924 (0.767, 1.081)	46.2%	4	1.201 (0.891, 1.511)	81.3%
4^th^ quintile	8	1.087 (0.991, 1.184)	00.0%	2	0.993 (0.816, 1.169)	50.7%	4	1.208 (1.047, 1.370)	27.6%
5^th^ quintile	8	1.156 (1.008, 1.304)	40.0%	2	1.016 (0.810, 1.222)	62.8%	4	1.091 (0.898, 1.284)	41.3%
**Overall**	**13**	**1.083 (1.032, 1.135)**	**17.6%**	**9**	**0.960 (0.896, 1.023)**	**26.1%**	**5**	**1.181 (1.088, 1.275)**	**50.7%**
**Liver**									
1^st^ tertile		Ref.			Ref.			Ref.	
2^nd^ tertile	0	No data	-	1	0.940 (0.585, 1.295)	-	1	1.970 (1.060, 2.880)	-
3^rd^ tertile	0	No data	-	1	0.700 (0.410, 0.990)	-	1	1.840 (0.960, 2.720)	-
1^st^ quartile		Ref.			Ref.			Ref.	-
2^nd^ quartile	0	No data	-	0	No data	-	0	No data	-
3^rd^ quartile	0	No data	-	0	No data	-	0	No data	-
4^th^ quartile	0	No data	-	0	No data	-	0	No data	-
1^st^ quintile		Ref.			Ref.			Ref.	
2^nd^ quintile	2	1.377 (1.000, 1.755)	00.0%	1	1.000 (0.750, 1.250)	-	1	1.130 (0.740, 1.520)	-
3^rd^ quintile	2	1.481 (1.076, 1.887)	00.0%	1	1.010 (0.755, 1.265)	-	1	1.110 (0.725, 1.495)	-
4^th^ quintile	2	1.458 (1.054, 1.863)	00.0%	1	0.950 (0.695, 1.205)	-	1	1.160 (0.750, 1.570)	-
5^th^ quintile	2	1.662 (1.202, 2.122)	00.0%	1	0.860 (0.620, 1.100)	-	1	1.090 (0.710, 1.470)	-
**Overall**	**2**	**1.481 (1.276, 1.685)**	**00.0%**	**2**	**0.916 (0.806, 1.025)**	**00.0%**	**2**	**1.196 (0.998, 1.394)**	**8.4%**
**Colon**									
1^st^ tertile		Ref.			Ref.			Ref.	
2^nd^ tertile	4	1.181 (0.777, 1.585)	51.8%	4	0.954 (0.775, 1.133)	00.0%	2	0.937 (0.769, 1.105)	77.1%
3^rd^ tertile	4	1.218 (0.819, 1.617)	24.6%	4	1.061 (0.870, 1.252)	00.0%	2	1.657 (0.310, 3.004)	49.7%
1^st^ quartile		Ref.			Ref.			Ref.	
2^nd^ quartile	9	1.102 (0.998, 1.205)	00.0%	8	0.932 (0.736, 1.129)	63.1%	7	1.093 (0.971, 1.215)	00.0%
3^rd^ quartile	9	1.086 (0.979, 1.193)	2.8%	8	0.938 (0.730, 1.147)	66.2%	7	1.006 (0.796, 1.216)	64.5%
4^th^ quartile	9	1.107 (0.938, 1.277)	50.0%	8	0.943 (0.634, 1.252)	83.8%	7	1.124 (0.793, 1.454)	82.2%
1^st^ quintile		Ref.			Ref.			Ref.	
2^nd^ quintile	8	0.993 (0.920, 1.065)	00.0%	3	0.967 (0.905, 1.029)	00.0%	5	1.071 (0.935, 1.207)	49.7%
3^rd^ quintile	8	0.980 (0.903, 1.056)	00.0%	3	0.954 (0.890, 1.017)	00.0%	5	1.012 (0.921, 1.103)	00.0%
4^th^ quintile	8	1.128 (1.043, 1.214)	00.0%	3	0.915 (0.834, 0.996)	28.4%	5	1.100 (1.001, 1.198)	00.0%
5^th^ quintile	8	1.178 (1.077, 1.279)	00.0%	3	0.924 (0.859, 0.990)	00.0%	5	1.152 (1.044, 1.260)	00.0%
**Overall**	**21**	**1.073 (1.034, 1.112)**	**17.3%**	**15**	**0.928 (0.874, 0.981)**	**56.6%**	**14**	**1.073 (1.018, 1.127)**	**47.6%**
**Rectum**									
1^st^ tertile		Ref.			Ref.			Ref.	
2^nd^ tertile	4	1.561 (1.158, 1.964)	00.0%	3	0.858 (0.569, 1.147)	40.9%	2	0.994 (0.850, 1.138)	00.0%
3^rd^ tertile	4	1.429 (0.775, 2.084)	46.0%	3	0.902 (0.693, 1.110)	00.0%	2	1.279 (0.389, 2.169)	73.1%
1^st^ quartile		Ref.			Ref.			Ref.	
2^nd^ quartile	9	1.081 (0.946, 1.217)	00.0%	4	0.930 (0.723, 1.137)	00.0%	6	1.113 (0.864, 1.362)	34.6%
3^rd^ quartile	9	1.021 (0.888, 1.154)	00.0%	4	0.910 (0.712, 1.109)	00.0%	6	1.188 (0.790, 1.585)	70.1%
4^th^ quartile	9	1.064 (0.843, 1.285)	49.4%	4	0.928 (0.717, 1.138)	00.0%	6	1.190 (0.870, 1.509)	48.1%
1^st^ quintile		Ref.			Ref.			Ref.	
2^nd^ quintile	6	1.067 (0.892, 1.242)	39.1%	3	0.904 (0.813, 0.994)	00.0%	5	1.051 (0.859, 1.242)	34.9%
3^rd^ quintile	6	1.173 (1.029, 1.316)	00.0%	3	0.849 (0.695, 1.002)	59.6%	5	1.005 (0.763, 1.246)	59.8%
4^th^ quintile	6	1.143 (0.994, 1.293)	00.0%	3	0.848 (0.757, 0.939)	3.2%	5	1.168 (1.000, 1.335)	00.0%
5^th^ quintile	6	1.180 (0.992, 1.367)	17.3%	3	0.845 (0.675, 1.016)	64.8%	5	1.062 (0.819, 1.305)	45.5%
**Overall**	**19**	**1.103 (1.045, 1.161)**	**13.1%**	**10**	**0.879 (0.839, 0.919)**	**00.0%**	**13**	**1.083 (1.002, 1.163)**	**43.3%**
**Colorectum**									
1^st^ tertile		Ref.			Ref.			Ref.	
2^nd^ tertile	12	1.102 (0.656, 1.547)	94.5%	8	0.868 (0.412, 1.323)	83.0%	4	1.070 (0.931, 1.208)	00.0%
3^rd^ tertile	12	1.319 (0.958, 1.680)	64.0%	8	1.031 (0.460, 1.601)	86.0%	4	1.709 (1.116, 2.303)	74.1%
1^st^ quartile		Ref.			Ref.			Ref.	
2^nd^ quartile	20	0.980 (0.871, 1.090)	53.7%	11	0.908 (0.808, 1.007)	00.0%	15	1.003 (0.900, 1.106)	27.9%
3^rd^ quartile	20	1.065 (0.993, 1.137)	00.0%	11	0.858 (0.744, 0.972)	21.4%	15	0.949 (0.871, 1.027)	00.0%
4^th^ quartile	20	1.177 (1.003, 1.350)	73.7%	11	0.885 (0.749, 1.022)	32.6%	15	1.283 (1.129, 1.437)	38.6%
1^st^ quintile		Ref.			Ref.			Ref.	
2^nd^ quintile	12	1.008 (0.956, 1.060)	00.0%	4	0.933 (0.868, 0.998)	00.0%	7	1.071 (1.009, 1.133)	00.0%
3^rd^ quintile	12	1.043 (0.975, 1.110)	18.1%	4	0.922 (0.854, 0.989)	00.0%	7	1.055 (0.989, 1.121)	00.0%
4^th^ quintile	12	1.109 (1.034, 1.184)	25.0%	4	0.863 (0.797, 0.928)	00.0%	7	1.151 (1.059, 1.243)	00.0%
5^th^ quintile	12	1.148 (1.067, 1.229)	51.0%	4	0.835 (0.749, 0.921)	23.9%	7	1.139 (1.068, 1.210)	00.0%
**Overall**	**44**	**1.088 (1.034, 1.143)**	**84.9%**	**23**	**0.862 (0.793, 0.931)**	**77.2%**	**26**	**1.081 (1.043, 1.119)**	**32.5%**

### Red meat

According to Table 1, high levels of red meat consumption were significantly associated with an increased risk of esophageal, pancreatic, liver, colon, rectal, and colorectal cancers (P<0.05). However, there was no statistically significant association between high levels of red meat consumption and stomach cancer risk (P>0.05).

### Fish

Table 1 indicates that high levels of fish consumption were significantly associated with a decreased risk of colon, rectal, and colorectal cancers (P<0.05). However, there was no statistically significant association between high levels of fish consumption and the risk of esophageal, stomach, pancreatic, and liver cancers (P>0.05).

### Processed meat

Table 1 shows that high levels of processed meat consumption were significantly associated with an increased risk of pancreatic, colon, rectal, and colorectal cancers (P<0.05). However, there was no statistically significant association between high levels of processed meat consumption and the risk of stomach, liver, and esophageal cancers (P>0.05).

### Processed fish

One study [25] (not included in Table 1) reported a non-significant association between high levels of processed fish consumption and a decreased risk of small intestine cancer (OR = 0.904; 95% CI: 0.678, 1.131). Two additional studies [26, 27] (not included in Table 1) reported a non-significant association between high levels of processed fish consumption and a decreased risk of stomach cancer (OR = 0.875; 95% CI: 0.615, 1.136). Another single study [28] (not included in Table 1) reported a non-significant association between high levels of processed fish consumption and a decreased risk of colon cancer (OR = 0.837; 95% CI: 0.641, 1.033).

The between-study heterogeneity was low for most of the overall estimates as shown in Table 1. The results of Begg’s and Egger’s tests did not reveal significant publication bias except for a few cases, which are reported in Table 2. However, the Trim-and-fill analysis did not significantly alter the results.

**Table 2:** The results of Begg’s and Egger’s tests (P-values) for assessing the possibility of publication bias in terms of gastrointestinal (GI) cancers.

	Red meat		Fish		Processed meat	
GI cancers	Begg’s Test	Egger’s test	Begg’s Test	Egger’s test	Begg’s Test	Egger’s test
Esophagus	0.714	0.872	0.805	0.862	0.702	0.586
Stomach	0.696	0.389	0.077	0.088	0.155	0.229
Pancreas	0.839	0.697	0.598	0.609	0.162	0.177
Liver	1.000	0.726	0.039	0.083	0.188	0.001
Colon	0.029	0.004	0.142	0.995	0.618	0.987
Rectum	0.004	0.003	0.915	0.629	0.410	0.139
Colorectum	0.001	0.011	0.377	0.530	0.378	0.453

**Fig 2:**
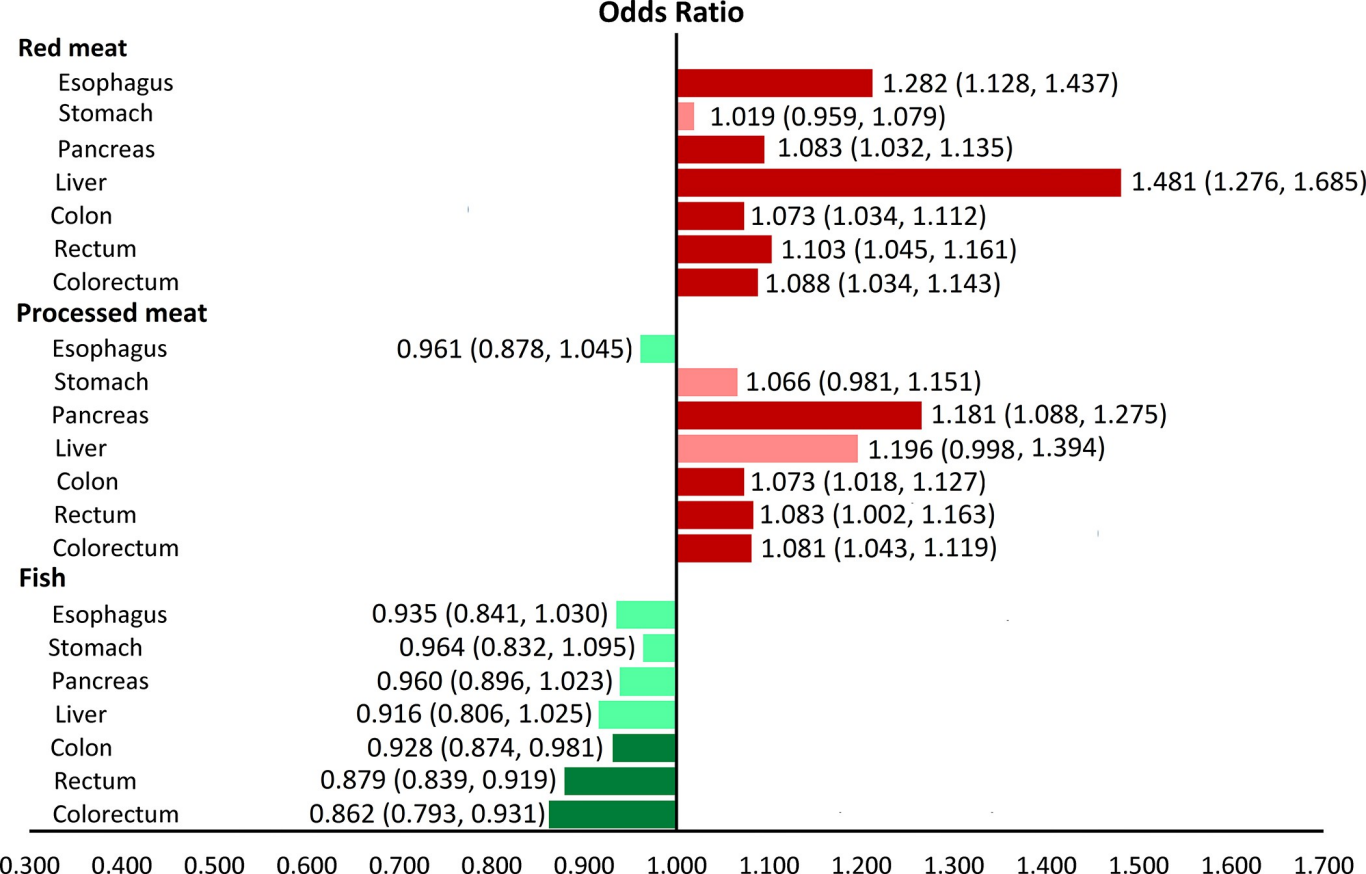
The associations [95% CIs] between gastrointestinal (GI) cancers and red meat, fish, and processed meat consumption in a single view. Protective factors are shown in green (dark green, significant; light green, non-significant), and risk factors are shown in red (dark red, significant; light red, non-significant).

Fig 2 provides a comprehensive summary of the associations between GI cancers and consumption of red meat, fish, and processed meat. The figure shows that consuming high levels of red meat was found to significantly increase the risk of esophageal, pancreatic, liver, colon, rectal, and colorectal cancers. Consuming high levels of processed meat was also found to significantly increase the risk of pancreatic, colon, rectal, and colorectal cancers. Conversely, consuming high levels of fish was associated with a significant reduction in the risk of pancreatic, colon, rectal, and colorectal cancers.

## Discussion

The meta-analysis found that high levels of red meat consumption were significantly associated with an increased risk of esophageal, pancreatic, liver, colon, rectal, and colorectal cancers, which confirms previous findings. In contrast, high levels of fish consumption were significantly associated with a decreased risk of colon, rectal, and colorectal cancers. However, the study did not find a statistically significant association between high levels of fish consumption and the risk of esophageal, stomach, pancreatic, and liver cancers. High levels of processed meat consumption were significantly associated with an increased risk of pancreatic, colon, rectal, and colorectal cancers, which is consistent with previous studies. The results suggest that reducing red and processed meat consumption and increasing fish consumption may have a protective effect against GI cancers. It is important to note that the study did not find a significant association between high levels of processed fish consumption and the risk of GI cancers. The low between-study heterogeneity and the absence of significant publication bias strengthen the validity of the findings. The comprehensive summary provided by Fig 2 confirms the associations between different types of meat consumption and GI cancer risk, which can be useful for public health recommendations and interventions. Overall, the study provides important insights into the role of diet in GI cancer prevention and highlights the need for further research in this area.

Our findings indicate that the consumption of red meat and processed meat is significantly associated with almost all major GI cancers. There are several mechanisms that can explain the impact of these types of meat on GI cancer risk. First, it is important to note that high-temperature cooking of red meat, such as grilling, barbecuing, or frying, can produce heterocyclic aromatic amines and polycyclic aromatic hydrocarbons. Many of these substances can generate DNA adducts, which can induce mutations and initiate carcinogenesis, thereby increasing the risk of GI cancers [29, 30]. Secondly, it is worth noting that N-glycolylneuraminic acid (Neu5Gc), a non-human sialic acid sugar, is highly enriched in red meat. Neu5Gc can bind to the cell membrane and interact with circulating anti-Neu5Gc antibodies, potentially causing inflammation and an immunological response that produces reactive oxygen species and accompanying inflammation. This can contribute to DNA damage, tumor promotion, and ultimately increase the risk of cancer, including GI cancers [30, 31]. Third, heme iron, which is present in red meat, has been shown to have several adverse effects on the body. It can cause cytotoxicity, boost epithelial cell proliferation, induce lipid peroxidation, create free radicals and DNA adducts, and catalyze the creation of N-nitroso compounds, all of which can contribute to the development of GI cancers [32, 33].

Our findings indicate a negative association between fish consumption and the risk of GI cancers. Oxidative stress and inflammation are major risk factors for chronic diseases, including cancer. Fatty fish, in particular, contain high levels of omega-3 fatty acids, which are polyunsaturated fatty acids (PUFAs). Omega-3 fatty acids have been shown to regulate the antioxidant signaling pathway, inhibit cell proliferation, and potentially lower oxidative stress by altering the inflammatory response [34, 35].

This meta-analysis provides evidence of associations between GI cancers and red and processed meat and fish consumption. However, cancer development is a complex process influenced by a combination of genetic, behavioral, and environmental factors [2]. Therefore, it is crucial to consider these risk and protective factors holistically, rather than in isolation. The likelihood of cancer increases when risk factors outweigh protective ones and vice versa [36]. In this context, while this study highlights the role of diet, particularly red and processed meat and fish consumption, in GI cancers, it is important to consider their impact alongside other influential factors for effective cancer prevention strategies.

Qin et al. conducted an umbrella review in 2022 to examine the role of dietary factors, including red meat and processed meat, in the development of esophageal cancer. Their findings showed that a higher intake of red meat and processed meat was positively associated with an increased risk of esophageal cancer [13]. In 2020, Poorolajal et al. conducted a meta-analysis to investigate the association between 14 behavioral and nutritional factors, including consumption of red meat and fish, and the development of stomach cancer. Their findings showed that a higher intake of fish was associated with a lower risk of stomach cancer, while a higher intake of red meat was associated with an increased risk of stomach cancer [4]. In 2022, Liu et al. conducted an umbrella review to investigate the role of dietary factors, including meat consumption, in the incidence of stomach cancer. Their findings indicated that high consumption of processed meat was associated with an increased incidence rate of stomach cancer [11]. In 2022, Gianfredi et al. conducted an umbrella review to investigate the role of dietary factors and patterns, including red meat, processed meat, poultry, and fish, in the development of pancreatic cancer. Their analysis showed that a high intake of red meat was associated with a higher risk of pancreatic cancer [9]. In 2023, Kim et al. conducted a meta-analysis to investigate the association between the consumption of red, processed, and white meat and the risk of pancreatic cancer. Their analysis revealed that a high intake of both red meat and white meat was associated with an increased risk of pancreatic cancer [10]. In 2023, Qin et al. conducted an umbrella review to investigate the association between dietary factors, including red meat consumption, and pancreatic cancer risk. Their findings revealed a positive association between a higher intake of red meat and the incidence of pancreatic cancer [12]. In 2022, Yu et al. conducted a meta-analysis to investigate the relationship between meat intake and the risk of hepatocellular carcinoma. Their findings indicated that consumption of red meat was not associated with an increased risk of the disease [14]. In 2014, Luo et al. conducted a meta-analysis to investigate the relationship between meat consumption and hepatocellular carcinoma. Their analysis showed that a higher intake of fish was significantly associated with a reduced risk of hepatocellular carcinoma. On the other hand, a higher intake of red meat and processed meat was not found to be associated with an increased risk of hepatocellular carcinoma [5]. In 2022, Gao et al. conducted a meta-analysis to investigate the association between poultry and fish intake and pancreatic cancer risk. Their findings suggested that high consumption of poultry intake may increase the risk of pancreatic cancer, while there was no significant link found between fish consumption and pancreatic cancer risk [8]. In 2022, Alegria-Lertxundi et al. conducted a systematic review to assess the potential role of dairy foods, fish, white meat, and eggs in the prevention of colorectal cancer. Their analysis indicated that the evidence for the association between fish, white meat, eggs, and colorectal cancer was not as strong [7]. While some studies suggest that more research is needed to strengthen the evidence, other studies have found significant associations between consumption of red and processed meat and increased risk of certain GI cancers, such as esophageal, stomach, and pancreatic cancer. However, it is still important to note that the available evidence is not yet sufficient to draw a definitive conclusion, and further research is needed to better understand the relationship between dietary factors and cancer risk and to make more specific recommendations for preventive measures.

Our study has several limitations. First, as with any meta-analysis based on observational studies, our findings suggest associations, not causation. Observational studies cannot definitively prove that red/processed meat consumption causes GI cancers or that fish consumption prevents them. Residual confounding variables, even after careful selection criteria, may still influence the observed relationships. Second, publication bias is a potential concern. Studies with statistically significant findings are more likely to be published than those with null results. This could have skewed our results towards stronger associations. Third, we excluded studies that did not categorize meat/fish consumption into tertiles, quartiles, or quintiles. While this ensured a standardized analysis, it may have introduced selection bias by omitting potentially relevant data. Finally, the inherent heterogeneity of dietary patterns and variations in study methodologies across different populations limit the generalizability of our findings. Future research that considers these factors and explores potential dose-response relationships would be valuable.

Our study acknowledges limitations but employed a rigorous search strategy to maximize the identification of relevant studies on the association between red/processed meat, fish consumption, and major GI cancers. This comprehensive analysis, encompassing various GI cancers, strengthens prior research and offers valuable insights for public health and future research. It highlights the importance of dietary patterns in GI cancer prevention, supporting existing recommendations to limit red and processed meat intake while increasing fish consumption. These findings can be used to improve public health. We can educate people about healthy portion sizes for meat and encourage them to choose fish proteins through campaigns and clear food labels. Additionally, policies that make healthy choices more affordable and accessible, like supporting fish producers and including fish on school menus, can give this a further boost.

## Conclusion

This meta-analysis of observational studies provides evidence indicating that a high consumption of red and processed meat is associated with an increased risk of several types of GI cancers, while a high consumption of fish is linked with a decreased risk of certain GI cancers. However, it is crucial to note that these findings do not prove causation and further research is necessary to confirm these associations and identify the potential underlying mechanisms involved. Nevertheless, the results emphasize the significance of considering dietary habits in the prevention of GI cancers and suggest that reducing the intake of red and processed meats while increasing fish consumption may have potential health benefits in lowering the risk of GI cancers. These findings have the potential to inform the development of effective public health interventions aimed at reducing the incidence of GI cancers.

## Supporting information

S1 Checklist (DOCX)

S1 Table Characteristics of the included studies (sorted by authors’ names). (DOCX)

S1 Fig Forrest plot of the association between gastrointestinal cancers and red and processed meat and fish consumption. (DOCX)

S1 Dataset (XLS)
