# Supplementary material for: The association between major gastrointestinal cancers and red and processed meat and fish consumption: A systematic review and meta-analysis of the observational studies
Source: PLoS One. 2024 Jun 26;19(6):e0305994. doi: 10.1371/journal.pone.0305994 (PMC11207151; doi:10.1371/journal.pone.0305994)
Supplement: S1 Table — (DOCX) [file pone.0305994.s002.docx]

**S1 Table: Summary of included studies** ^1-95^

| Row | 1^st^ author | Country | Sex | Age | GI cancers | Meat products | Classification | Study design | Effect | Sample | Quality |
| --- | --- | --- | --- | --- | --- | --- | --- | --- | --- | --- | --- |
|  | Aglago 2020 | Europe | Both | - | Colon, rectum, colorectum | Fish | Quintiles | Cohort | Hazard Ratio | 476,160 | ******** |
|  | Al Rajabi 2021 | Canada | Both | 50.0 | Colorectum | Meat, processed meat | Quartiles | Cohort | Hazard Ratio | 26,218 | ****** |
|  | Alegria~ 2020 | Spain | Both | 61.3 | Colorectum | Meat, fish | Tertiles | Case-control | Odds Ratio | 616 | ****** |
|  | Andersen 2002 | Denmark | Both | 65.5 | Pancreas | Meat | Quintiles | Case-control | Odds Ratio | 867 | ******** |
|  | Archambault 2021 | USA | Both | <50 | Colorectum | Meat, processed meat | Quartiles | Case-control | Odds Ratio | 777,545 | ****** |
|  | Aune 2009 | Uruguay | Both | 62.3 | Esophagus, stomach, pancreas,  colon, rectum | Meat, processed meat | Tertiles | Case-control | Odds Ratio | 5,571 | ******* |
|  | Austin 2007 | USA | Both | 56.2 | Colorectum | Meat | Tertiles | Case-control | Odds Ratio | 725 | ******* |
|  | Batlle 2016 | Spain | Both | 64.4 | Colorectum | Meat, processed meat | Tertiles | Case-control | Odds Ratio | 4,766 | ******* |
|  | Bidoli 1992 | Italy | Both | 57.5 | Colon, rectum | Meat, fish | Tertiles | Case-control | Odds Ratio | 947 | ******** |
|  | Boeing 1991 | Germany | Both | 32-80 | Stomach | Processed meat, processed fish | Tertiles | Case-control | Risk Ratio | 722 | ******* |
|  | Breuer~ 2001 | Germany | Both | 63.8 | Colorectum | Meat | Quintiles | Case-control | Odds Ratio | 542 | ****** |
|  | Budhathoki 2019 | Brazil | Both | 62.1 | Colorectum | Meat, processed meat, fish | Quartiles | Cohort | Odds Ratio | 705 | ******** |
|  | Butler 2003 | USA | Both | 40-84 | Colon | Meat | Quintiles | Case-control | Odds Ratio | 1,658 | ******** |
|  | Chan 2007 | USA | Both | - | Pancreas | Meat | Quartiles | Case-control | Odds Ratio | 2,233 | ****** |
|  | Chao 2005 | USA | Both | 63.0 | Colon, rectum, colorectum | Meat, processed meat | Quintiles | Cohort | Risk Ratio | 1,667 | ******* |
|  | Chen 2015 | Canada | Both | 20-74 | Colorectum | Meat | Quintiles | Case-control | Odds Ratio | 1,179 | ****** |
|  | Chiu 2003 | China | Both | 30-74 | Colon | Fish | Quartiles | Case-control | Odds Ratio | 2,483 | **** |
|  | Chiu 2004 | China | Both | 57.4 | Colorectum | Meat, processed meat, fish | Quartiles | Case-control | Odds Ratio | 372 | ********* |
|  | Chun 2015 | Korea | Both | 20-80 | Colorectum | Meat, fish | Tertiles | Case-control | Odds Ratio | 266 | ****** |
|  | Cross 2007 | USA | Both | 50-71 | Esophagus, pancreas, stomach,  liver, colorectum | Meat, processed meat | Quintiles | Cohort | Hazard Ratio | 567,169 | ***** |
|  | Cross 2010 | USA | Both | 50-71 | Colon, rectum, colorectum | Meat, processed meat | Quintiles | Cohort | Hazard Ratio | 300,948 | ****** |
|  | Cross 2011 | USA | Both | 50-71 | Esophagus, stomach | Meat, processed meat | Quintiles | Cohort | Hazard Ratio | 303,156 | ****** |
|  | Cross 2014 | USA | Both | 18-74 | Colorectum | Meat, processed meat | Quartiles | Case-control | Odds Ratio | 262 | ******* |
|  | Daniel 2011 | USA | Both | 50-71 | Esophagus, stomach, pancreas,  liver, small intestine, colon, rectum | Fish | Quintiles | Cohort | Hazard Ratio | 492,186 | ****** |
|  | De Stefani 1997 | Uruguay | Both | 25-84 | Colon, rectum, colorectum | Meat, processed meat | Quartiles | Case-control | Risk Ratio | 750 | ******* |
|  | De Stefani 1999 | Uruguay | Both | 40-89 | Esophagus | Meat, processed meat, fish | Tertiles | Case-control | Odds Ratio | 330 | ******** |
|  | De Stefani 2001 | Uruguay | Both | 32.35 | Stomach | Meat, processed meat | Tertiles | Case-control | Odds Ratio | 405 | ******* |
|  | De Stefani 2004 | Uruguay | Both | 30-89 | Stomach | Meat, fish | Tertiles | Case-control | Odds Ratio | 2,080 | ******* |
|  | Engeset 2007 | Norway | Both | 51.3 | Colon | Fish, processed fish | Tertiles | Cohort | Risk Ratio | 68,517 | ***** |
|  | English 2004 | Australia | Both | 25-75 | Colon, rectum, colorectum | Meat, processed meat, fish | Quartiles | Cohort | Hazard Ratio | 41,528 | ***** |
|  | Epplein 2014 | China | Both | 40-74 | Stomach | Meat, processed meat | Tertiles | Cohort | Odds Ratio | 61,491 | ******* |
|  | Evans 2002 | UK | Both | 67.0 | Colorectum | Meat | Quartiles | Case-control | Odds Ratio | 1,024 | ****** |
|  | Fan 2008 | China | Both | 55.3 | Esophagus | Meat, fish | Tertiles | Cohort | Hazard Ratio | 18,244 | ****** |
|  | Fernandez 1996 | Italy | Both | 62.0 | Colon, rectum, colorectum | Meat | Quartiles | Case-control | Risk Ratio | 3,350 | ******* |
|  | Fernandez 1997 | Italy | Both | <75 | Colorectum | Meat | Tertiles | Case-control | Risk Ratio | 4,463 | ******* |
|  | Ferro 2019 | USA | Both | - | Stomach | Meat, processed meat | Tertiles | Case-control | Odds Ratio | 47,720 | ***** |
|  | Ferrucci 2009 | USA | Both | - | Colorectum | Meat, processed meat, fish | Quartiles | Cross-sectional | Odds Ratio | 807 | ******* |
|  | Ferrucci 2012 | USA | Both | 55-74 | Colon, rectum, colorectum | Meat, processed meat | Quartiles | Cohort | Odds Ratio | 154,952 | ****** |
|  | Flood 2003 | USA | Both | 61.9 | Colorectum | Meat, processed meat | Quintiles | Cohort | Risk Ratio | 45,496 | ****** |
|  | Franceschi 1997 | Italy | Both | 62.0 | Colorectum | Meat, processed meat, fish | Quintiles | Case-control | Odds Ratio | 6,107 | ******* |
|  | Fredrikson 1995 | Swedish | Both | 30-75 | Colon | Fish | Quartiles | Case-control | Odds Ratio | 713 | ****** |
|  | Freedman 2010 | USA | Both | 50-71 | Liver | Meat | Quintiles | Cohort | Hazard Ratio | 495,006 | ****** |
|  | Ghadirian 2010 | Canada | Both | 35-79 | Pancreas | Meat, fish | Quartiles | Case-control | Odds Ratio | 686 | ****** |
|  | Gilsing 2015 | Netherlands | Both | 61.3 | Colon, rectum, colorectum | Meat, processed meat | 4th Quartiles | Cohort | Hazard Ratio | 10,609 | ****** |
|  | Giovannucci 1992 | USA | Both | 40-75 | Colorectum | Meat | Quintiles | Cohort | Risk Ratio | 51,529 | **** |
|  | He 2012 | USA | Both | 50-76 | Pancreas | Fish | Tertiles | Cohort | Hazard Ratio | 66,616 | **** |
|  | Heinen 2009 | Netherlands | Both | 55-69 | Pancreas | Meat, processed meat, fish | Quartiles | Cohort | Risk Ratio | 120,852 | ***** |
|  | Hsing 1998 | USA | Both | ≥35 | Colon, colorectum | Meat, fish | Quintiles | Cohort | Risk Ratio | 17,633 | ***** |
|  | Hu 2008 | Canada | Both | 20-76 | Stomach, pancreas, colon, rectum | Meat, processed meat, fish | Quartiles | Case-control | Odds Ratio | 14,771 | ****** |
|  | Inoue-Choi 2011 | USA | Both | 55-69 | Pancreas | Meat | Quintiles | Cohort | Hazard Ratio | 41,836 | ***** |
|  | Iscovich 1992 | Argentina | Both | 65.0 | Colon | Processed meat, fish | Quartiles | Case-control | Odds Ratio | 330 | **** |
|  | Iswarya 2016 | India | Both | 17-78 | Colorectum | Fish | Tertiles | Case-control | Odds Ratio | 94 | ***** |
|  | Ito 2003 | Japan | Both | 48.6 | Stomach | Meat, processed meat | Quartiles | Cohort | Odds Ratio | 36,490 | ******* |
|  | Jansen 2013 | USA | Both | - | Pancreas | Meat | Quartiles | Case-control | Odds Ratio | 1,331 | ****** |
|  | Ji 1995 | China | Both | 30-74 | Pancreas | Fish | Quartiles | Case-control | Odds Ratio | 2,003 | ***** |
|  | Jiao 2015 | USA | Both | 10.5 | Pancreas | Meat, processed meat | Quintiles | Cohort | Hazard Ratio | 2,193 | ***** |
|  | Joshi 2015 | USA | Both | 58.6 | Colon, rectum, colorectum | Meat, processed meat | Quintiles | Case-control | Odds Ratio | 6,854 | ******** |
|  | Kato 1997 | USA | Both | 34-65 | Colorectum | Meat, fish | Quartiles | Cohort | Risk Ratio | 14,727 | ****** |
|  | Kimura 2007 | Japan | Both | 20-74 | Colorectum | Meat, processed meat, fish | Quintiles | Case-control | Odds Ratio | 1,575 | ******* |
|  | Kobayashi 2004 | Japan | Both | 40-69 | Colon, rectum | Fish | Quartiles | Cohort | Risk Ratio | 88,658 | ********* |
|  | Larsson 2006 | Sweden | Both | - | Stomach | Meat, processed meat, fish | Tertiles | Cohort | Hazard Ratio | 66,651 | ****** |
|  | Lazarevic 2010 | Serbia | Both | 50-85 | Stomach | Fish | Tertiles | Case-control | Odds Ratio | 306 | ****** |
|  | Lee 1989 | Singapore | Both | - | Colon, rectum | Meat, fish | Tertiles | Case-control | Odds Ratio | 628 | ******* |
|  | Levi 1999 | Switzerland | Both | 58.0 | Colorectum | Meat, processed meat, fish | Tertiles | Case-control | Odds Ratio | 714 | ******* |
|  | Luchtenborg 2005 | Netherlands | Both | 55-69 | Colon, rectum | Meat, fish | Quartiles | Case-cohort | Rate Ratio | 2,948 | ******** |
|  | Ma 2019 | USA | Both | 30-75 | Liver | Processed meat, fish | Tertiles | Cohort | Hazard Ratio | 142,857 | ****** |
|  | Marchand 2002 | USA | Both | - | Colon, rectum | Meat | Tertiles | Case-control | Odds Ratio | 1,454 | ***** |
|  | Mehta 2019 | USA | Both | 35-74 | Colorectum | Meat, processed meat | Quartiles | Cohort | Risk Ratio | 50,884 | ******* |
|  | Momenyan 2017 | Iran | Both | >18 | Colorectum | Meat, fish | Tertiles | Case-control | Odds Ratio | 305 | ******* |
|  | Navarro 2003 | Argentina | Both | 60.7 | Colorectum | Meat, fish | Tertiles | Case-control | Odds Ratio | 853 | ******* |
|  | Nayak 2009 | India | Both | 55.8 | Colorectum | Meat, fish | Tertiles | Case-control | Odds Ratio | 432 | ******* |
|  | Ngoan 2002 | Fukuoka | Both | - | Stomach | Processed meat, fish, processed fish | Tertiles | Cohort | Risk Ratio | 13,250 | ****** |
|  | Nöthlings 2005 | USA | Both | 60.0 | Pancreas | Meat, processed meat, fish | Quintiles | Cohort | Risk Ratio | 364,483 | ****** |
|  | Oh 2005 | Korea | Both | 53.3 | Colorectum | Meat, fish | Tertiles | Case-control | Odds Ratio | 270 | ******* |
|  | Ollberding 2012 | USA | Both | 45-75 | Colorectum | Meat | Quintiles | Cohort | Risk Ratio | 165,717 | ****** |
|  | Pietinen 1999 | Finland | Both | 57.1 | Colorectum | Meat, processed meat, fish | Quartiles | Cohort | Risk Ratio | 27,111 | ****** |
|  | Polesel 2009 | Italy | Both | 63.0 | Pancreas | Meat | Quintiles | Case-control | Odds Ratio | 978 | ******* |
|  | Robertson 2005 | USA | Both | 61.0 | Colorectum | Meat, processed meat, fish | Quartiles | Cohort | Risk Ratio | 1,520 | ******* |
|  | Rosato 2013 | Europe | Both | 40.0 | Colorectum | Meat, processed meat | Tertiles | Case-control | Odds Ratio | 1,690 | ******* |
|  | Rosato 2017 | Italy | Both | - | Colon, rectum | Processed meat | Tertiles | Case-control | Odds Ratio | 10,549 | ******* |
|  | Salamat 2020 | Iran | Both | - | Esophagus | Meat, fish | Tertiles | Cohort | Odds Ratio | 4,710 | ******** |
|  | Sato 2006 | Japan | Both | 40-64 | Colon, rectum, colorectum | Meat | Quartiles | Cohort | Risk Ratio | 51,921 | ******* |
|  | Sinha 2005 | Korea | Both | 55-74 | Colon, rectum | Meat | Quintiles | Case-control | Odds Ratio | 38,513 | ******** |
|  | Stefani 2012 | Uruguay | Both | 30-89 | Esophagus | Meat, processed meat | Tertiles | Case-control | Odds Ratio | 2,254 | ********* |
|  | Sugawara 2009 | Japan | Both | 40-79 | Colon, rectum, colorectum | Fish | Quartiles | Cohort | Hazard Ratio | 39,498 | ****** |
|  | Takachi 2011 | Japan | Both | 45-74 | Colon, rectum | Meat, processed meat | Quintiles | Cohort | Hazard Ratio |  | ***** |
|  | Takezaki 2001 | China | Both | 40-79 | Esophagus, stomach | Fish | Quartiles | Case-control | Odds Ratio | 719 | ******** |
|  | Taunk 2016 | USA | Both | 50-71 | Pancreas | Meat | Quintiles | Cohort | Hazard Ratio | 322,846 | ******* |
|  | Vecchia 1990 | Italy | Both | 54.0 | Pancreas | Fish | Tertiles | Case-control | Risk Ratio | 1,336 | ******** |
|  | Vulcan 2017 | Sweden | Both | - | Colon, rectum, colorectum | Meat, processed meat, fish | Quintiles | Cohort | Hazard Ratio | 28,098 | ******** |
|  | Wada 2017 | Japan | Both | ≥35 | Colon, rectum, colorectum | Meat, processed meat | Quartiles | Cohort | Risk Ratio | 30,331 | ******** |
|  | Wang 2012 | China | Both | 30-79 | Stomach | Meat, fish | Tertiles | Case-control | Odds Ratio | 771 | ****** |
|  | Zamani 2013 | Iran | Both | 62.3 | Stomach | Meat | Quartiles | Case-control | Odds Ratio | 837 | ***** |
|  | Zaridze 1993 | Russia | Both | - | Colorectum | Meat, fish | Quartiles | Case-control | Odds Ratio | 234 | ****** |
|  | Zheng 1993 | USA | Both | ≥35 | Pancreas | Meat, fish | Quartiles | Cohort | Risk Ratio | 17,818 | ******* |

**References**

1. Aglago EK, Bray F, Zotor F, Slimani N, Chajès V, Huybrechts I, et al. Temporal trends in food group availability and cancer incidence in Africa: an ecological analysis. Public Health Nutr. 2019; 22:2569-80.

2. Al Rajabi A, Lo Siou G, Akawung AK, McDonald K, Price TR, Shen-Tu G, et al. Towards refining World Cancer Research Fund/American Institute for Cancer Research cancer prevention recommendations for red and processed meat intake: insights from Alberta's Tomorrow Project cohort. Br J Nutr. 2022; 127:607-18.

3. Alegria-Lertxundi I, Aguirre C, Bujanda L, Fernandez FJ, Polo F, Ordovas JM, et al. Food groups, diet quality and colorectal cancer risk in the Basque Country. World Journal of Gastroenterology. 2020; 26:4108-25.

4. Anderson KE, Sinha R, Kulldorff M, Gross M, Lang NP, Barber C, et al. Meat intake and cooking techniques: associations with pancreatic cancer. Mutat Res. 2002; 506-507:225-31.

5. Archambault AN, Lin Y, Jeon J, Harrison TA, Bishop DT, Brenner H, et al. Nongenetic Determinants of Risk for Early-Onset Colorectal Cancer. JNCI Cancer Spectr. 2021; 5.

6. Aune D, De Stefani E, Ronco A, Boffetta P, Deneo-Pellegrini H, Acosta G, et al. Meat consumption and cancer risk: a case-control study in Uruguay. Asian Pac J Cancer Prev. 2009; 10:429-36.

7. Austin GL, Adair LS, Galanko JA, Martin CF, Satia JA, Sandler RS. A diet high in fruits and low in meats reduces the risk of colorectal adenomas. J Nutr. 2007; 137:999-1004.

8. Bidoli E, Franceschi S, Talamini R, Barra S, La Vecchia C. Food consumption and cancer of the colon and rectum in north-eastern Italy. Int J Cancer. 1992; 50:223-9.

9. Boeing H, Frentzel-Beyme R, Berger M, Berndt V, Göres W, Körner M, et al. Case-control study on stomach cancer in Germany. Int J Cancer. 1991; 47:858-64.

10. Breuer-Katschinski B, Nemes K, Marr A, Rump B, Leiendecker B, Breuer N, et al. Colorectal adenomas and diet - A case-control study. Digestive Diseases and Sciences. 2001; 46:86-95.

11. Budhathoki S, Iwasaki M, Yamaji T, Hamada GS, Miyajima NT, Zampieri JC, et al. Doneness preferences, meat and meat-derived heterocyclic amines intake, and N-acetyltransferase 2 polymorphisms: association with colorectal adenoma in Japanese Brazilians. European Journal of Cancer Prevention. 2020; 29:7-14.

12. Butler LM, Sinha R, Millikan RC, Martin CF, Newman B, Gammon MD, et al. Heterocyclic amines, meat intake, and association with colon cancer in a population-based study. Am J Epidemiol. 2003; 157:434-45.

13. Chan JM, Wang F, Holly EA. Pancreatic cancer, animal protein and dietary fat in a population-based study, San Francisco Bay Area, California. Cancer Causes Control. 2007; 18:1153-67.

14. Chao A, Thun MJ, Connell CJ, McCullough ML, Jacobs EJ, Flanders WD, et al. Meat consumption and risk of colorectal cancer. Jama. 2005; 293:172-82.

15. Chen Z, Wang PP, Woodrow J, Zhu Y, Roebothan B, McLaughlin JR, et al. Dietary patterns and colorectal cancer: results from a Canadian population-based study. Nutr J. 2015; 14:8.

16. Chiu BC, Gapstur SM. Changes in diet during adult life and risk of colorectal adenomas. Nutr Cancer. 2004; 49:49-58.

17. Chiu BC, Ji BT, Dai Q, Gridley G, McLaughlin JK, Gao YT, et al. Dietary factors and risk of colon cancer in Shanghai, China. Cancer Epidemiol Biomarkers Prev. 2003; 12:201-8.

18. Chun YJ, Sohn SK, Song HK, Lee SM, Youn YH, Lee S, et al. Associations of colorectal cancer incidence with nutrient and food group intakes in korean adults: a case-control study. Clin Nutr Res. 2015; 4:110-23.

19. Cross AJ, Ferrucci LM, Risch A, Graubard BI, Ward MH, Park Y, et al. A large prospective study of meat consumption and colorectal cancer risk: an investigation of potential mechanisms underlying this association. Cancer Res. 2010; 70:2406-14.

20. Cross AJ, Freedman ND, Ren J, Ward MH, Hollenbeck AR, Schatzkin A, et al. Meat consumption and risk of esophageal and gastric cancer in a large prospective study. Am J Gastroenterol. 2011; 106:432-42.

21. Cross AJ, Leitzmann MF, Gail MH, Hollenbeck AR, Schatzkin A, Sinha R. A prospective study of red and processed meat intake in relation to cancer risk. PLoS Med. 2007; 4:e325.

22. Cross AJ, Major JM, Rothman N, Sinha R. Urinary 1-methylhistidine and 3-methylhistidine, meat intake, and colorectal adenoma risk. Eur J Cancer Prev. 2014; 23:385-90.

23. Daniel CR, Cross AJ, Graubard BI, Hollenbeck AR, Park Y, Sinha R. Prospective investigation of poultry and fish intake in relation to cancer risk. Cancer Prev Res (Phila). 2011; 4:1903-11.

24. De Stefani E, Correa P, Boffetta P, Deneo-Pellegrini H, Ronco AL, Mendilaharsu M. Dietary patterns and risk of gastric cancer: a case-control study in Uruguay. Gastric Cancer. 2004; 7:211–20.

25. De Stefani E, Deneopellegrini H, Mendilaharsu M, Ronco A. Meat intake, heterocyclic amines and risk of colorectal cancer. Int J Oncol. 1997; 10:573-80.

26. De Stefani E, Deneo-Pellegrini H, Mendilaharsu M, Ronco A. Diet and risk of cancer of the upper aerodigestive tract - I. Foods. Oral Oncology. 1999; 35:17-21.

27. De Stefani E, Ronco A, Brennan P, Boffetta P. Meat consumption and risk of stomach cancer in Uruguay: a case-control study. Nutr Cancer. 2001; 40:103-7.

28. Engeset D, Andersen V, Hjartaker A, Lund E. Consumption of fish and risk of colon cancer in the Norwegian Women and Cancer (NOWAC) study. British Journal of Nutrition. 2007; 98:576-82.

29. English DR, MacInnis RJ, Hodge AM, Hopper JL, Giles GG. Red meat, chicken and fish consumption and risk of colorectal cancer in the Melbourne Collaborative Cohort study. Cancer Epidemiology Biomarkers & Prevention. 2002; 11:1220S-1S.

30. Epplein M, Zheng W, Li H, Peek RM, Jr., Correa P, Gao J, et al. Diet, Helicobacter pylori strain-specific infection, and gastric cancer risk among Chinese men. Nutr Cancer. 2014; 66:550-7.

31. Evans RC, Fear S, Ashby D, Hackett A, Williams E, Van Der Vliet M, et al. Diet and colorectal cancer: an investigation of the lectin/galactose hypothesis. Gastroenterology. 2002; 122:1784-92.

32. Fan Y, Yuan JM, Wang R, Gao YT, Yu MC. Alcohol, tobacco, and diet in relation to esophageal cancer: the Shanghai Cohort Study. Nutr Cancer. 2008; 60:354-63.

33. Fernandez E, D'Avanzo B, Negri E, Franceschi S, La Vecchia C. Diet diversity and the risk of colorectal cancer in northern Italy. Cancer Epidemiol Biomarkers Prev. 1996; 5:433-6.

34. Fernandez E, La Vecchia C, D'Avanzo B, Negri E, Franceschi S. Risk factors for colorectal cancer in subjects with family history of the disease. Br J Cancer. 1997; 75:1381-4.

35. Ferro A, Rosato V, Rota M, Costa AR, Morais S, Pelucchi C, et al. Meat intake and risk of gastric cancer in the Stomach cancer Pooling (StoP) project. Int J Cancer. 2020; 147:45-55.

36. Ferrucci LM, Cross AJ, Graubard BI, Brinton LA, McCarty CA, Ziegler RG, et al. Intake of meat, meat mutagens, and iron and the risk of breast cancer in the Prostate, Lung, Colorectal, and Ovarian Cancer Screening Trial. Br J Cancer. 2009; 101:178-84.

37. Ferrucci LM, Sinha R, Huang WY, Berndt SI, Katki HA, Schoen RE, et al. Meat consumption and the risk of incident distal colon and rectal adenoma. Br J Cancer. 2012; 106:608-16.

38. Flood A, Velie EM, Sinha R, Chaterjee N, Lacey JV, Jr., Schairer C, et al. Meat, fat, and their subtypes as risk factors for colorectal cancer in a prospective cohort of women. Am J Epidemiol. 2003; 158:59-68.

39. Franceschi S, Favero A, La Vecchia C, Negri E, Conti E, Montella M, et al. Food groups and risk of colorectal cancer in Italy. Int J Cancer. 1997; 72:56-61.

40. Fredrikson M, Hardell L, Bengtsson N, Axelson O. Colon-cancer and dietary habits - a case-control study. Int J Oncol. 1995; 7:133-41.

41. Freedman ND, Cross AJ, McGlynn KA, Abnet CC, Park Y, Hollenbeck AR, et al. Association of meat and fat intake with liver disease and hepatocellular carcinoma in the NIH-AARP cohort. J Natl Cancer Inst. 2010; 102:1354-65.

42. Ghadirian P, Nkondjock A. Consumption of food groups and the risk of pancreatic cancer: a case-control study. J Gastrointest Cancer. 2010; 41:121-9.

43. Gilsing AM, Schouten LJ, Goldbohm RA, Dagnelie PC, van den Brandt PA, Weijenberg MP. Vegetarianism, low meat consumption and the risk of colorectal cancer in a population based cohort study. Sci Rep. 2015; 5:13484.

44. Giovannucci E, Stampfer MJ, Colditz G, Rimm EB, Willett WC. Relationship of diet to risk of colorectal adenoma in men. J Natl Cancer Inst. 1992; 84:91-8.

45. He K, Xun PC, Brasky TM, Gammon MD, Stevens J, White E. Types of Fish Consumed and Fish Preparation Methods in Relation to Pancreatic Cancer Incidence The VITAL Cohort Study. American Journal of Epidemiology. 2013; 177:152-60.

46. Heinen MM, Verhage BA, Goldbohm RA, van den Brandt PA. Meat and fat intake and pancreatic cancer risk in the Netherlands Cohort Study. Int J Cancer. 2009; 125:1118-26.

47. Hsing AW, McLaughlin JK, Chow WH, Schuman LM, Chien HTC, Gridley G, et al. Risk factors for colorectal cancer in a prospective study among US white men. International Journal of Cancer. 1998; 77:549-53.

48. Hu J, La Vecchia C, DesMeules M, Negri E, Mery L. Meat and fish consumption and cancer in Canada. Nutr Cancer. 2008; 60:313-24.

49. Inoue-Choi M, Flood A, Robien K, Anderson K. Nutrients, food groups, dietary patterns, and risk of pancreatic cancer in postmenopausal women. Cancer Epidemiol Biomarkers Prev. 2011; 20:711-4.

50. Iscovich JM, L'Abbé KA, Castelleto R, Calzona A, Bernedo A, Chopita NA, et al. Colon cancer in Argentina. I: Risk from intake of dietary items. Int J Cancer. 1992; 51:851-7.

51. Iswarya SK, Premarajan KC, Kar SS, Kumar SS, Kate V. Risk factors for the development of colorectal carcinoma: A case control study from South India. World J Gastrointest Oncol. 2016; 8:207-14.

52. Ito LS, Inoue M, Tajima K, Yamamura Y, Kodera Y, Hirose K, et al. Dietary factors and the risk of gastric cancer among Japanese women: a comparison between the differentiated and non-differentiated subtypes. Ann Epidemiol. 2003; 13:24-31.

53. Jansen RJ, Robinson DP, Frank RD, Stolzenberg-Solomon RZ, Bamlet WR, Oberg AL, et al. Meat-related mutagens and pancreatic cancer: null results from a clinic-based case-control study. Cancer Epidemiol Biomarkers Prev. 2013; 22:1336-9.

54. Ji BT, Chow WH, Gridley G, McLaughlin JK, Dai Q, Wacholder S, et al. Dietary factors and the risk of pancreatic cancer: a case-control study in Shanghai China. Cancer Epidemiol Biomarkers Prev. 1995; 4:885-93.

55. Jiao L, Stolzenberg-Solomon R, Zimmerman TP, Duan Z, Chen L, Kahle L, et al. Dietary consumption of advanced glycation end products and pancreatic cancer in the prospective NIH-AARP Diet and Health Study. Am J Clin Nutr. 2015; 101:126-34.

56. Joshi AD, Kim A, Lewinger JP, Ulrich CM, Potter JD, Cotterchio M, et al. Meat intake, cooking methods, dietary carcinogens, and colorectal cancer risk: findings from the Colorectal Cancer Family Registry. Cancer Med. 2015; 4:936-52.

57. Kato I, Akhmedkhanov A, Koenig K, Toniolo PG, Shore RE, Riboli E. Prospective study of diet and female colorectal cancer: the New York University Women's Health Study. Nutr Cancer. 1997; 28:276-81.

58. Kimura Y, Kono S, Toyomura K, Nagano J, Mizoue T, Moore MA, et al. Meat, fish and fat intake in relation to subsite-specific risk of colorectal cancer: The Fukuoka Colorectal Cancer Study. Cancer Sci. 2007; 98:590-7.

59. Kobayashi M, Tsubono Y, Otani T, Hanaoka T, Sobue T, Tsugane S. Fish, long-chain n-3 polyunsaturated fatty acids, and risk of colorectal cancer in middle-aged Japanese: the JPHC study. Nutr Cancer. 2004; 49:32-40.

60. Larsson SC, Bergkvist L, Wolk A. Processed meat consumption, dietary nitrosamines and stomach cancer risk in a cohort of Swedish women. Int J Cancer. 2006; 119:915-9.

61. Lazarevic K, Nagorni A, Rancic N, Milutinovic S, Stosic L, Ilijev I. Dietary factors and gastric cancer risk: hospital-based case control study. J buon. 2010; 15:89-93.

62. Lee HP, Gourley L, Duffy SW, Estève J, Lee J, Day NE. Colorectal cancer and diet in an Asian population--a case-control study among Singapore Chinese. Int J Cancer. 1989; 43:1007-16.

63. Levi F, Pasche C, La Vecchia C, Lucchini F, Franceschi S. Food groups and colorectal cancer risk. Br J Cancer. 1999; 79:1283-7.

64. Luchtenborg M, Weijenberg MP, de Goeij A, Wark PA, Brink M, Roemen G, et al. Meat and fish consumption, APC gene mutations and hMLH1 expression in colon and rectal cancer: a prospective cohort study (The Netherlands). Cancer Causes & Control. 2005; 16:1041-54.

65. Ma Y, Yang W, Li T, Liu Y, Simon TG, Sui J, et al. Meat intake and risk of hepatocellular carcinoma in two large US prospective cohorts of women and men. Int J Epidemiol. 2019; 48:1863-71.

66. Marchand LL, Hankin JH, Pierce LM, Rashmi Sinha, Nerurkar PV, Franke AA, et al. Well-done red meat, metabolic phenotypes and colorectal cancer in Hawaii. Mutation Research. 2002:205–14.

67. Mehta SS, Arroyave WD, Lunn RM, Park YM, Boyd WA, Sandler DP. A Prospective Analysis of Red and Processed Meat Consumption and Risk of Colorectal Cancer in Women. Cancer Epidemiol Biomarkers Prev. 2020; 29:141-50.

68. Momenyan S, Ghalane S, Sarvi F, Azizi R, Kabiri F. The Association between Lifestyle, Occupational, and Reproductive Factors and Colorectal Cancer Risk. Asian Pac J Cancer Prev. 2017; 18:2157-62.

69. Navarro A, Diaz MP, Munoz SE, Lantieri MJ, Eynard AR. Characterization of meat consumption and risk of colorectal cancer in Cordoba, Argentina. Nutrition. 2003; 19:7-10.

70. Nayak SP, Sasi MP, Sreejayan MP, Mandal S. A case-control study of roles of diet in colorectal carcinoma in a South Indian Population. Asian Pac J Cancer Prev. 2009; 10:565-8.

71. Ngoan LT, Mizoue T, Fujino Y, Tokui N, Yoshimura T. Dietary factors and stomach cancer mortality. British Journal of Cancer. 2002; 87:37-42.

72. Nothlings U, Wilkens LR, Murphy SP, Hankin JH, Henderson BE, Kolonel LN. Meat and fat intake as risk factors for pancreatic cancer: The Multiethnic Cohort Study. Jnci-Journal of the National Cancer Institute. 2005; 97:1458-65.

73. Oh SY, Ji HL, Dong KJ, Seung CH, Hyo JK. Relationship of nutrients and food to colorectal cancer risk in Koreans. Nutrition Research. 2005; 25:805-13.

74. Ollberding NJ, Wilkens LR, Henderson BE, Kolonel LN, Le Marchand L. Meat consumption, heterocyclic amines and colorectal cancer risk: the Multiethnic Cohort Study. Int J Cancer. 2012; 131:E1125-33.

75. Pietinen P, Malila N, Virtanen M, Hartman TJ, Tangrea JA, Albanes D, et al. Diet and risk of colorectal cancer in a cohort of Finnish men. Cancer Causes Control. 1999; 10:387-96.

76. Polesel J, Talamini R, Negri E, Bosetti C, Boz G, Lucenteforte E, et al. Dietary habits and risk of pancreatic cancer: an Italian case-control study. Cancer Causes Control. 2010; 21:493-500.

77. Robertson DJ, Sandler RS, Haile R, Tosteson TD, Greenberg ER, Grau M, et al. Fat, fiber, meat and the risk of colorectal adenomas. Am J Gastroenterol. 2005; 100:2789-95.

78. Rosato V, Bosetti C, Levi F, Polesel J, Zucchetto A, Negri E, et al. Risk factors for young-onset colorectal cancer. Cancer Causes Control. 2013; 24:335-41.

79. Rosato V, Tavani A, Negri E, Serraino D, Montella M, Decarli A, et al. Processed Meat and Colorectal Cancer Risk: A Pooled Analysis of Three Italian Case-Control Studies. Nutr Cancer. 2017; 69:732-8.

80. Salamat F, Semnani S, Honarvar MR, Fazel A, Roshandel G. 10-Year Trends in Dietary Intakes in the High- and Low-Risk Areas for Esophageal Cancer: A Population-Based Ecological Study in Northern Iran. Middle East J Dig Dis. 2020; 12:89-98.

81. Sato Y, Nakaya N, Kuriyama S, Nishino Y, Tsubono Y, Tsuji I. Meat consumption and risk of colorectal cancer in Japan: the Miyagi Cohort Study. Eur J Cancer Prev. 2006; 15:211-8.

82. Sinha R, Kulldorff M, Gunter MJ, Strickland P, Rothman N. Dietary benzo[a]pyrene intake and risk of colorectal adenoma. Cancer Epidemiol Biomarkers Prev. 2005; 14:2030-4.

83. Stefani ED, Correa P, Boffetta P, Deneo-Pellegrini H, Ronco AL, Mendilaharsu M. Dietary patterns and risk of gastric cancer: a case-control study in Uruguay. Gastric Cancer. 2004; 7:211–20.

84. Sugawara Y, Kuriyama S, Kakizaki M, Nagai M, Ohmori-Matsuda K, Sone T, et al. Fish consumption and the risk of colorectal cancer: the Ohsaki Cohort Study. Br J Cancer. 2009; 101:849-54.

85. Takachi R, Tsubono Y, Baba K, Inoue M, Sasazuki S, Iwasaki M, et al. Red meat intake may increase the risk of colon cancer in Japanese, a population with relatively low red meat consumption. Asia Pac J Clin Nutr. 2011; 20:603-12.

86. Takezaki T, Gao CM, Wu JZ, Ding JH, Liu YT, Zhang Y, et al. Dietary protective and risk factors for esophageal and stomach cancers in a low-epidemic area for stomach cancer in Jiangsu Province, China: comparison with those in a high-epidemic area. Jpn J Cancer Res. 2001; 92:1157-65.

87. Taunk P, Hecht E, Stolzenberg-Solomon R. Are meat and heme iron intake associated with pancreatic cancer? Results from the NIH-AARP diet and health cohort. Int J Cancer. 2016; 138:2172-89.

88. Vecchia CL, Negri E, Avanzo BD, Ferraroni M, Gramenzi A, Savoldelli R, et al. Medical History, diet and pancreatic cancer. Oncology. 1990; 47:463-6.

89. Vulcan A, Manjer J, Ericson U, Ohlsson B. Intake of different types of red meat, poultry, and fish and incident colorectal cancer in women and men: results from the Malmö Diet and Cancer Study. Food Nutr Res. 2017; 61:1341810.

90. Wada K, Oba S, Tsuji M, Tamura T, Konishi K, Goto Y, et al. Meat consumption and colorectal cancer risk in Japan: The Takayama study. Cancer Sci. 2017; 108:1065-70.

91. Wang J, Joshi AD, Corral R, Siegmund KD, Le Marchand L, Martinez ME, et al. Carcinogen metabolism genes, red meat and poultry intake, and colorectal cancer risk. International Journal of Cancer. 2012; 130:1898-907.

92. Zamani N, Hajifaraji M, Fazel-tabar Malekshah A, Keshtkar AA, Esmaillzadeh A, Malekzadeh R. A case-control study of the relationship between gastric cancer and meat consumption in Iran. Arch Iran Med. 2013; 16:324-9.

93. Zaridze D, Filipchenko V, Kustov V, Serdyuk V, Duffy S. DIET AND COLORECTAL-CANCER - RESULTS OF 2 CASE-CONTROL STUDIES IN RUSSIA. European Journal of Cancer. 1993; 29A:112-5.

94. Zheng W, McLaughlin JK, Gridley G, Bjelke E, Schuman LM, Silverman DT, et al. A cohort study of smoking, alcohol consumption, and dietary factors for pancreatic cancer (United States). Cancer Causes Control. 1993; 4:477-82.

95. Batlle Jd, Gracia-Lavedan E, Romaguera D, Mendez· M, Castaño-Vinyals G, Martín V, et al. Meat intake, cooking methods and doneness and risk of colorectal tumours in the Spanish multicase-control study (MCC-Spain). Eur J Nutr. 2016.
