# Supplementary material for: The association between major gastrointestinal cancers and red and processed meat and fish consumption: A systematic review and meta-analysis of the observational studies
Source: PLoS One. 2024 Jun 26;19(6):e0305994. doi: 10.1371/journal.pone.0305994 (PMC11207151; doi:10.1371/journal.pone.0305994)
Supplement: S1 Fig — (DOCX) [file pone.0305994.s003.docx]

**S1 Fig:** Forrest plots of the association between gastrointestinal cancers and red and processed meat and fish consumption


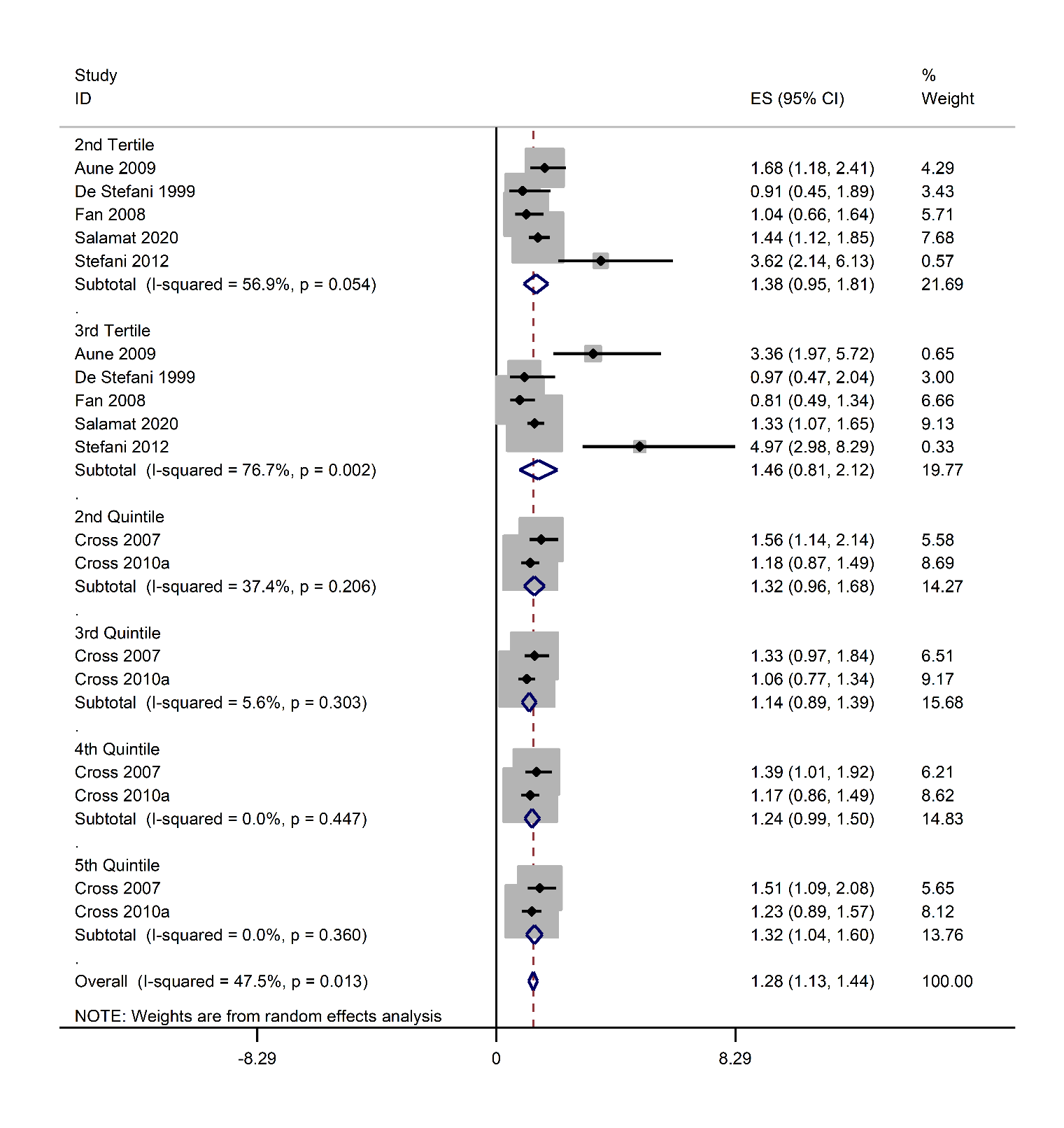


Forrest plot of the association between **esophageal** cancer and consumption of **red meat** classified as tertiles, quartiles, and quintiles

**
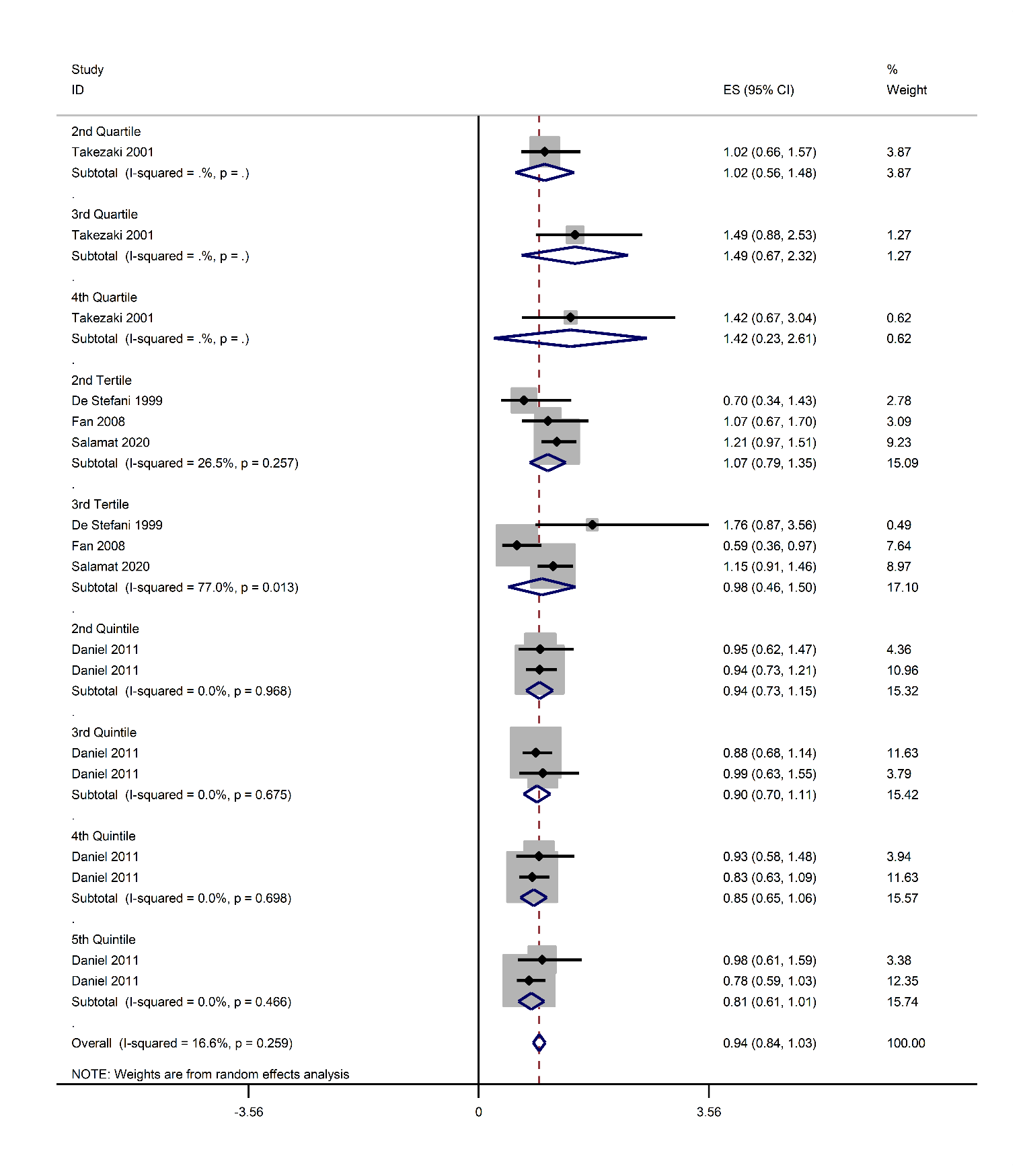
**

Forrest plot of the association between **esophageal** cancer and consumption of **fish** classified as tertiles, quartiles, and quintiles


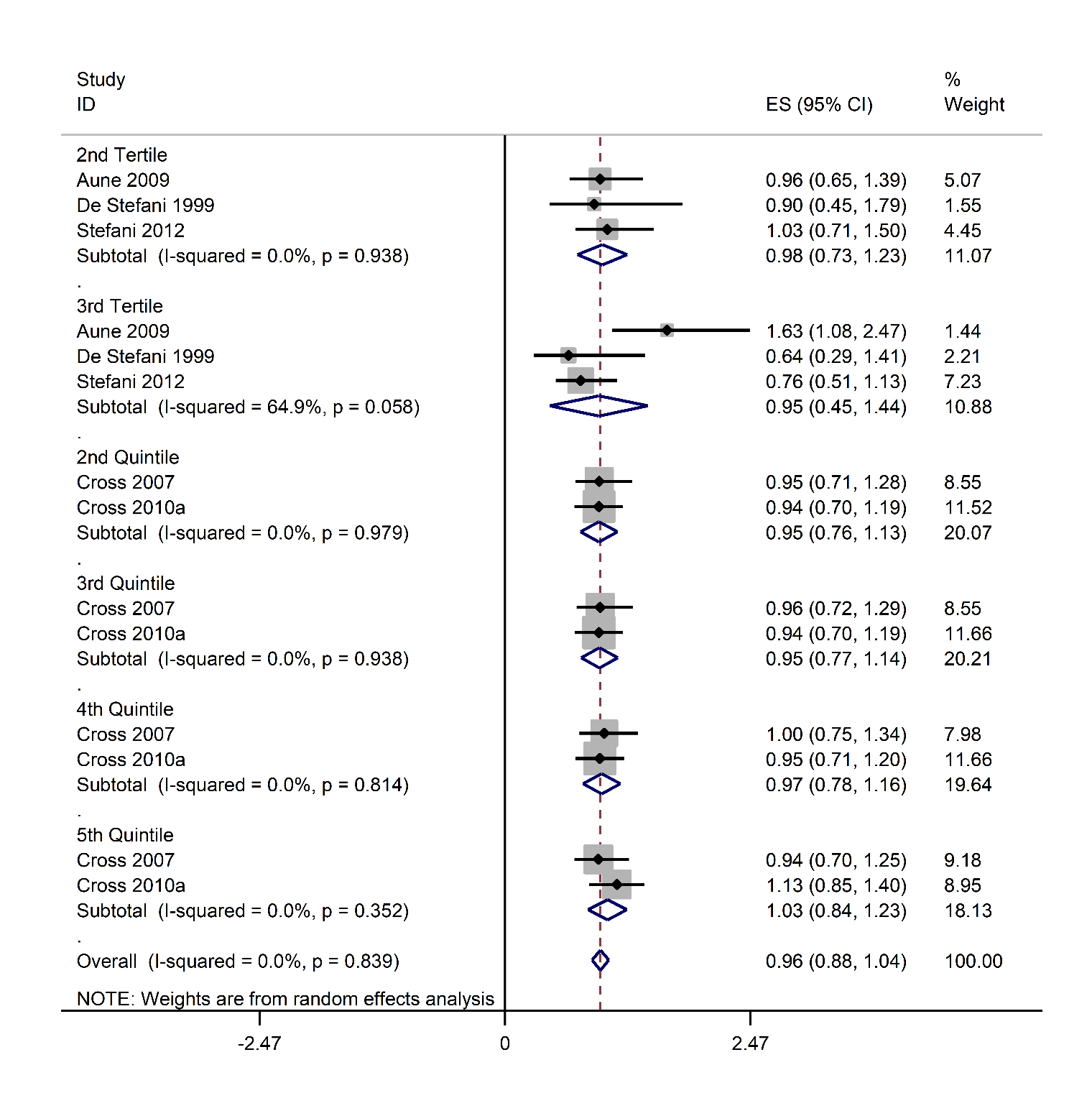


Forrest plot of the association between **esophageal** cancer and consumption of **processed** **meat** classified as tertiles, quartiles, and quintiles


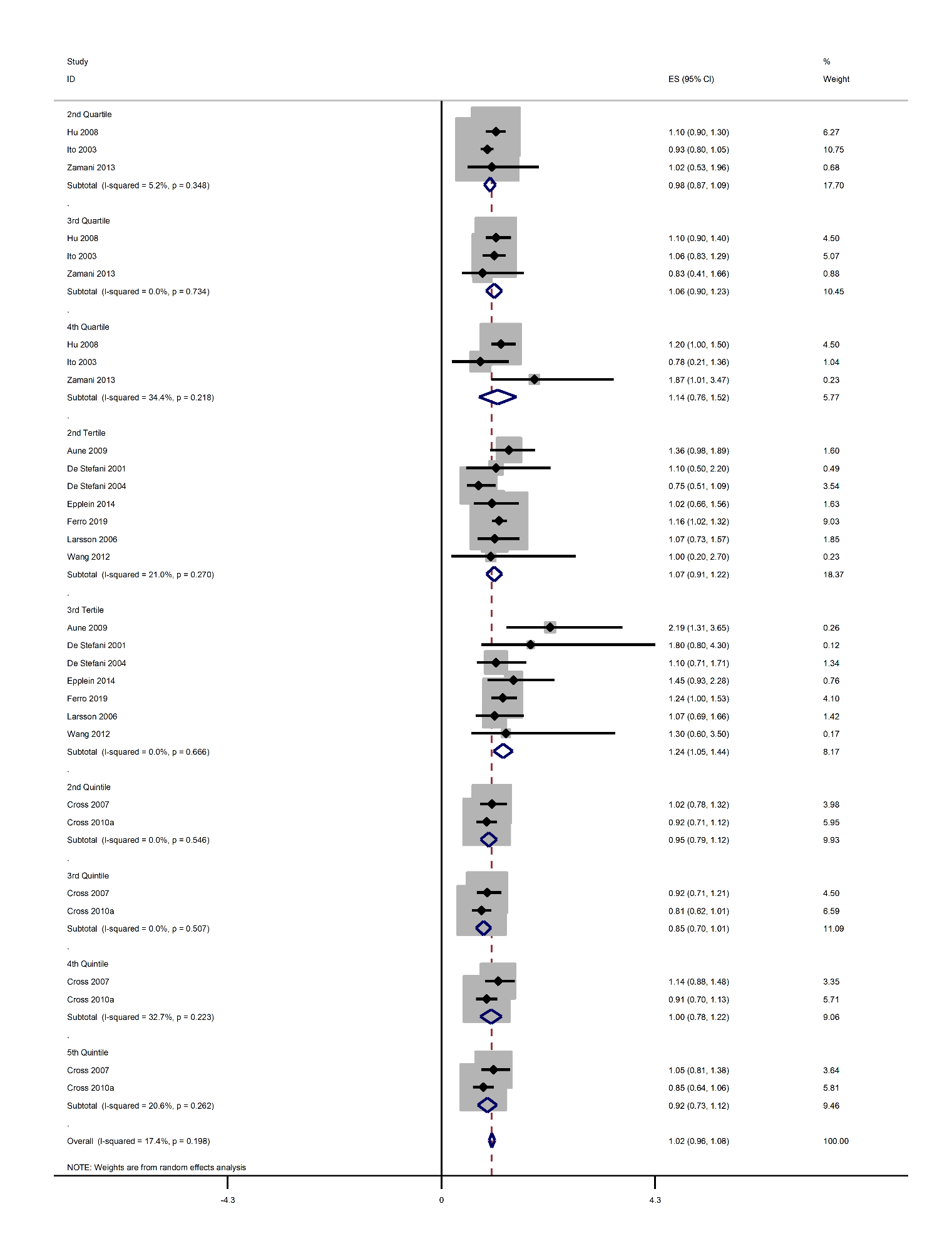


Forrest plot of the association between **stomach** cancer and consumption of **red meat** classified as tertiles, quartiles, and quintiles


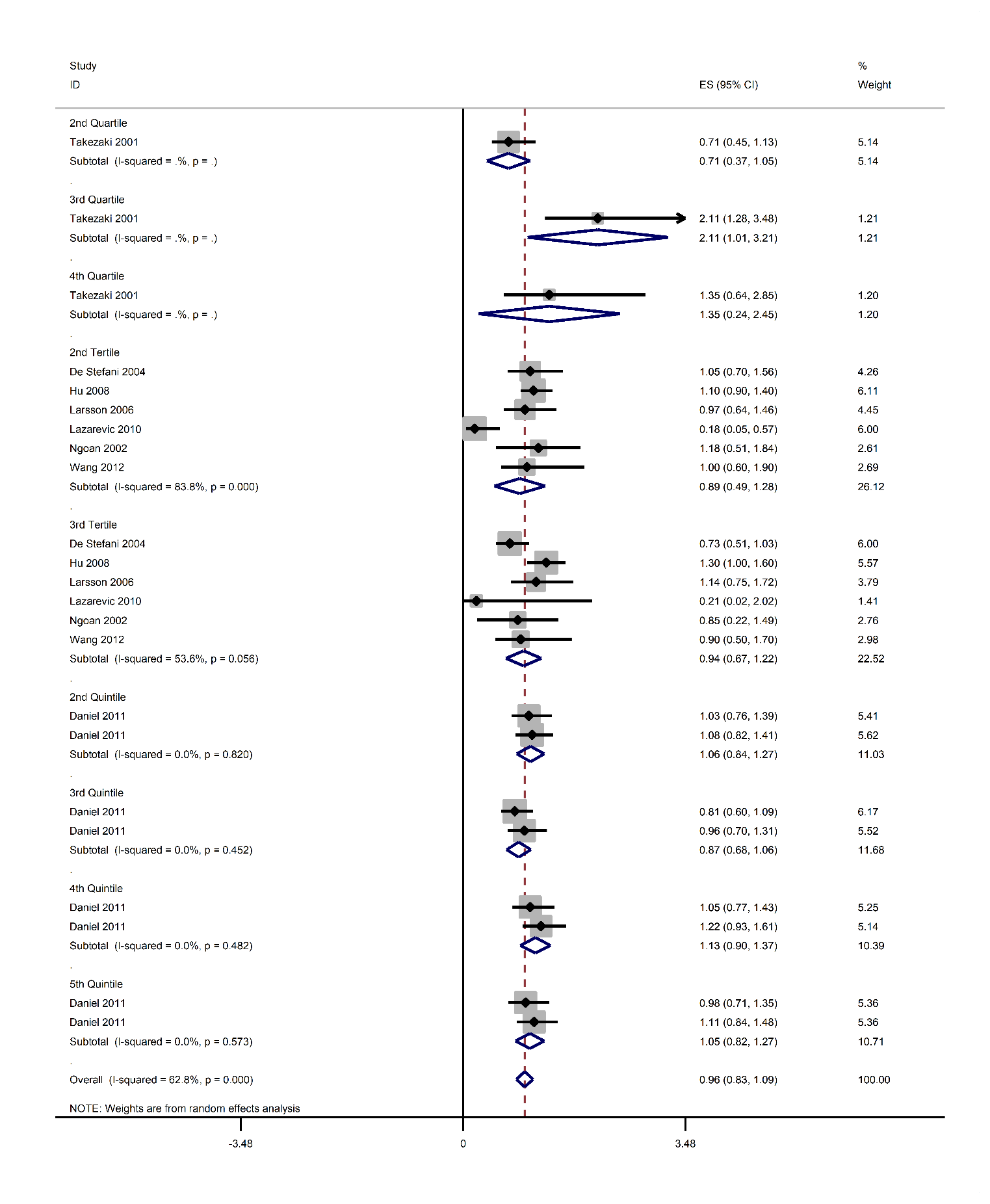


Forrest plot of the association between **stomach** cancer and consumption of **fish** classified as tertiles, quartiles, and quintiles


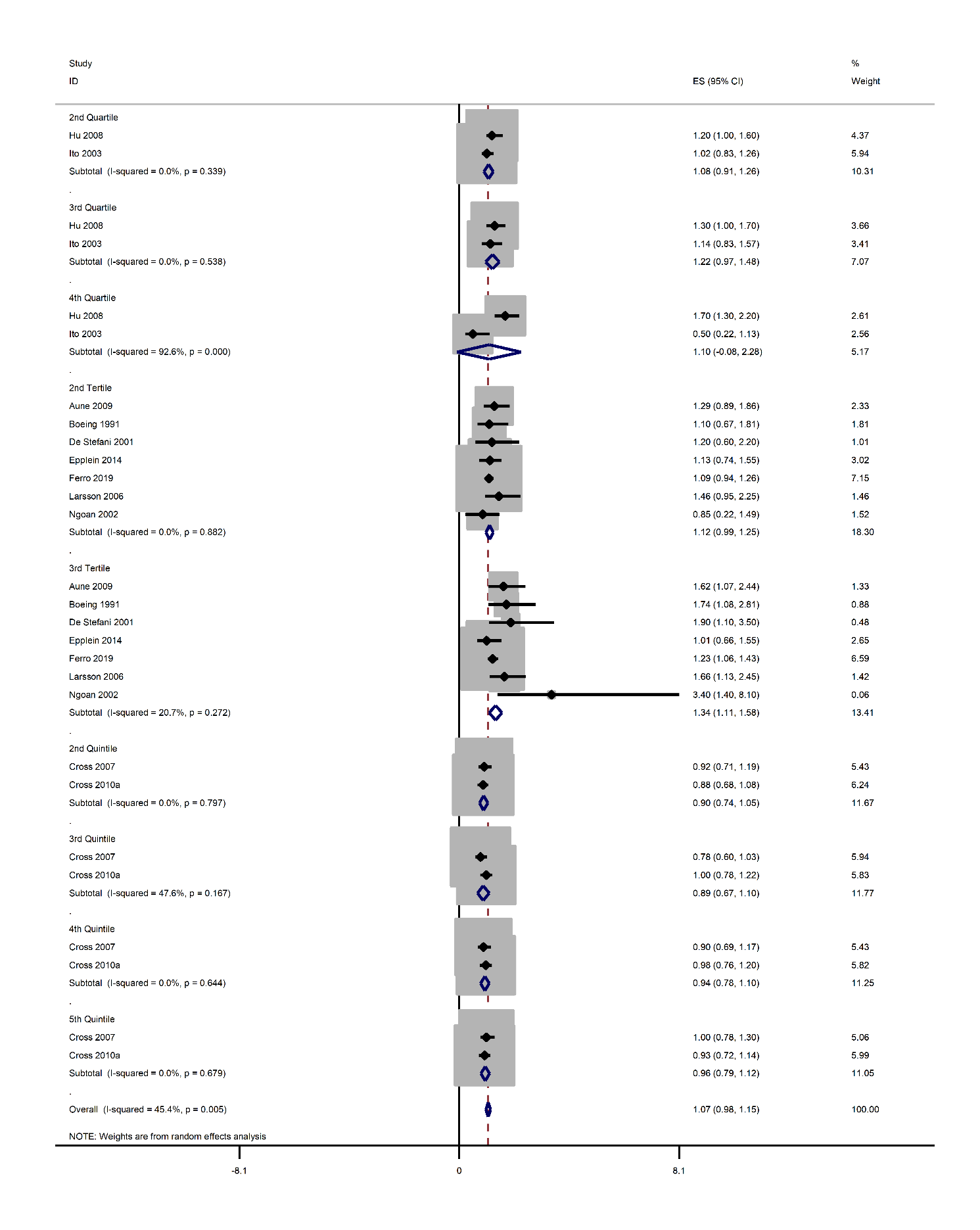


Forrest plot of the association between **stomach** cancer and consumption of **processed meat** classified as tertiles, quartiles, and quintiles

**
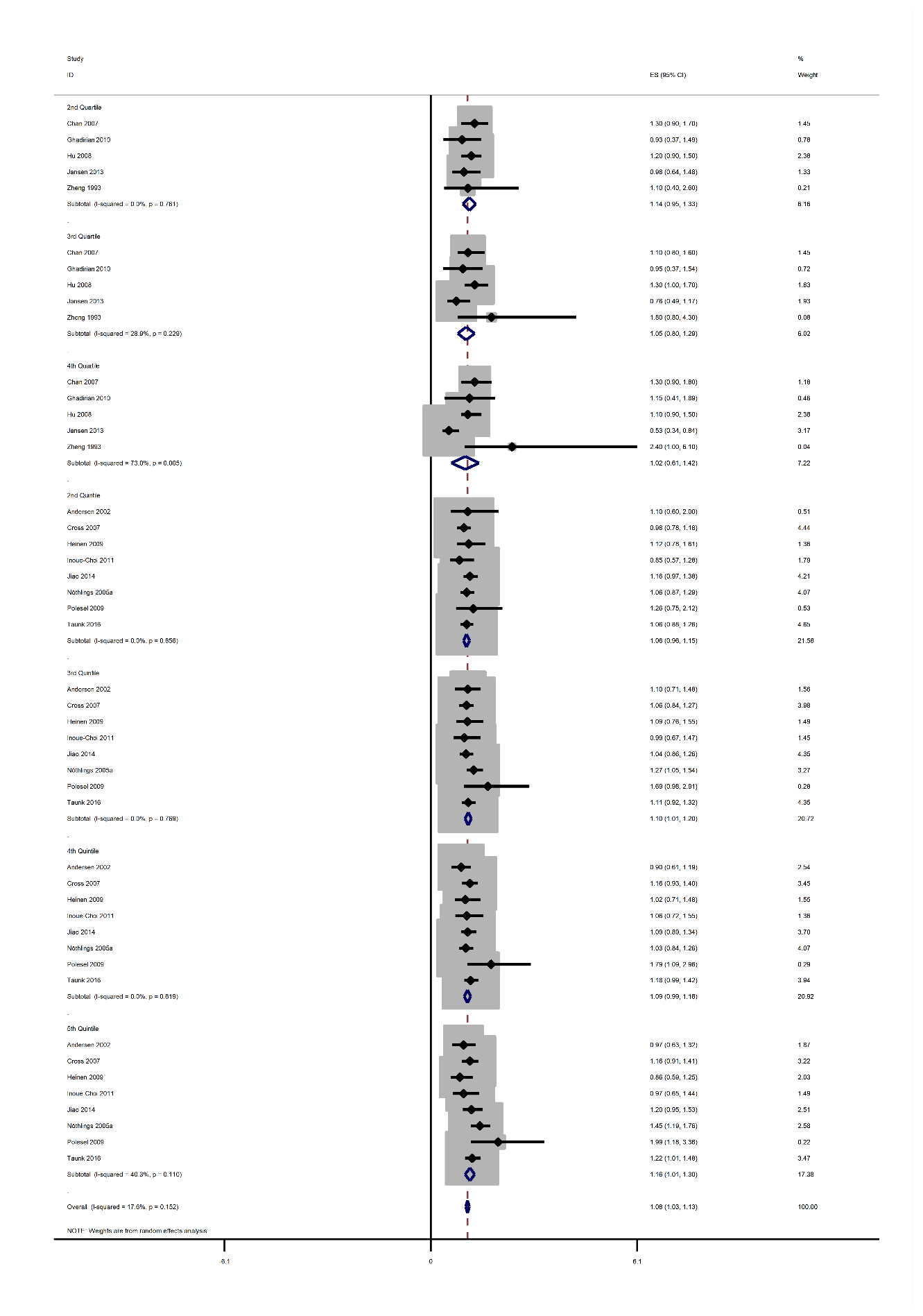
**

Forrest plot of the association between **pancreatic** cancer and consumption of **red meat** classified as tertiles, quartiles, and quintiles

**
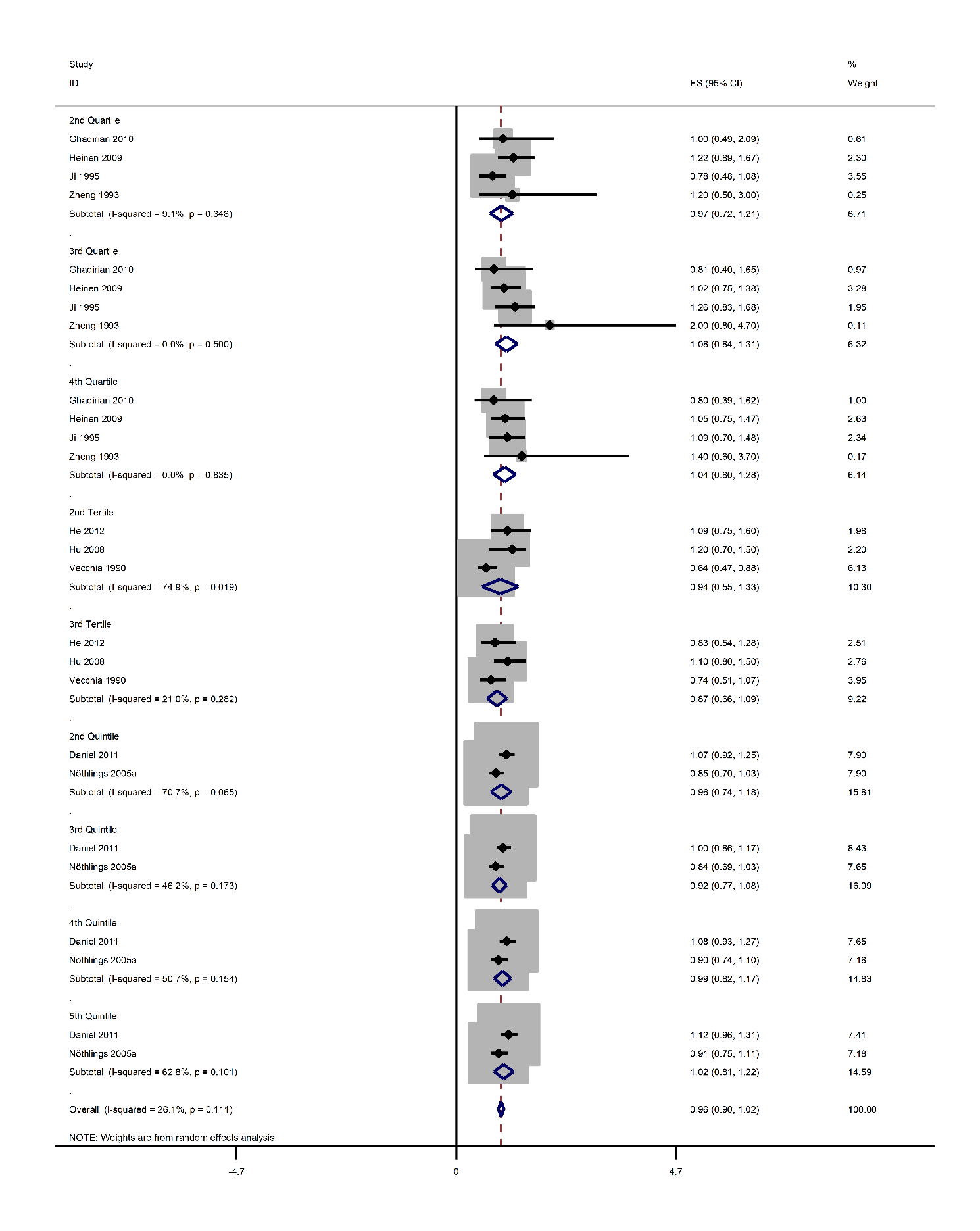
**

Forrest plot of the association between **pancreatic** cancer and consumption of **fish** classified as tertiles, quartiles, and quintiles

**
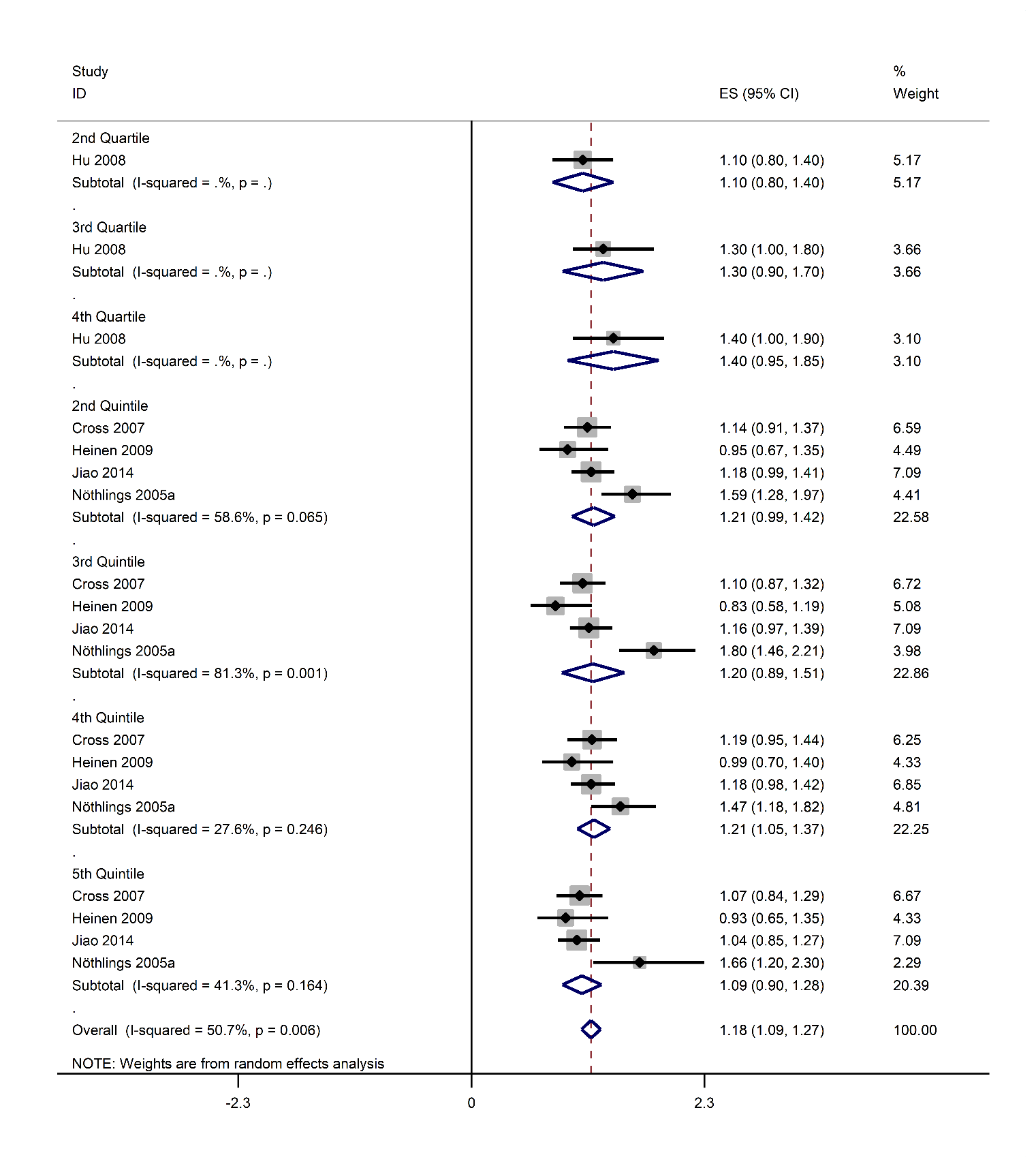
**

Forrest plot of the association between **pancreatic** cancer and consumption of **processed meat** classified as tertiles, quartiles, and quintiles

**
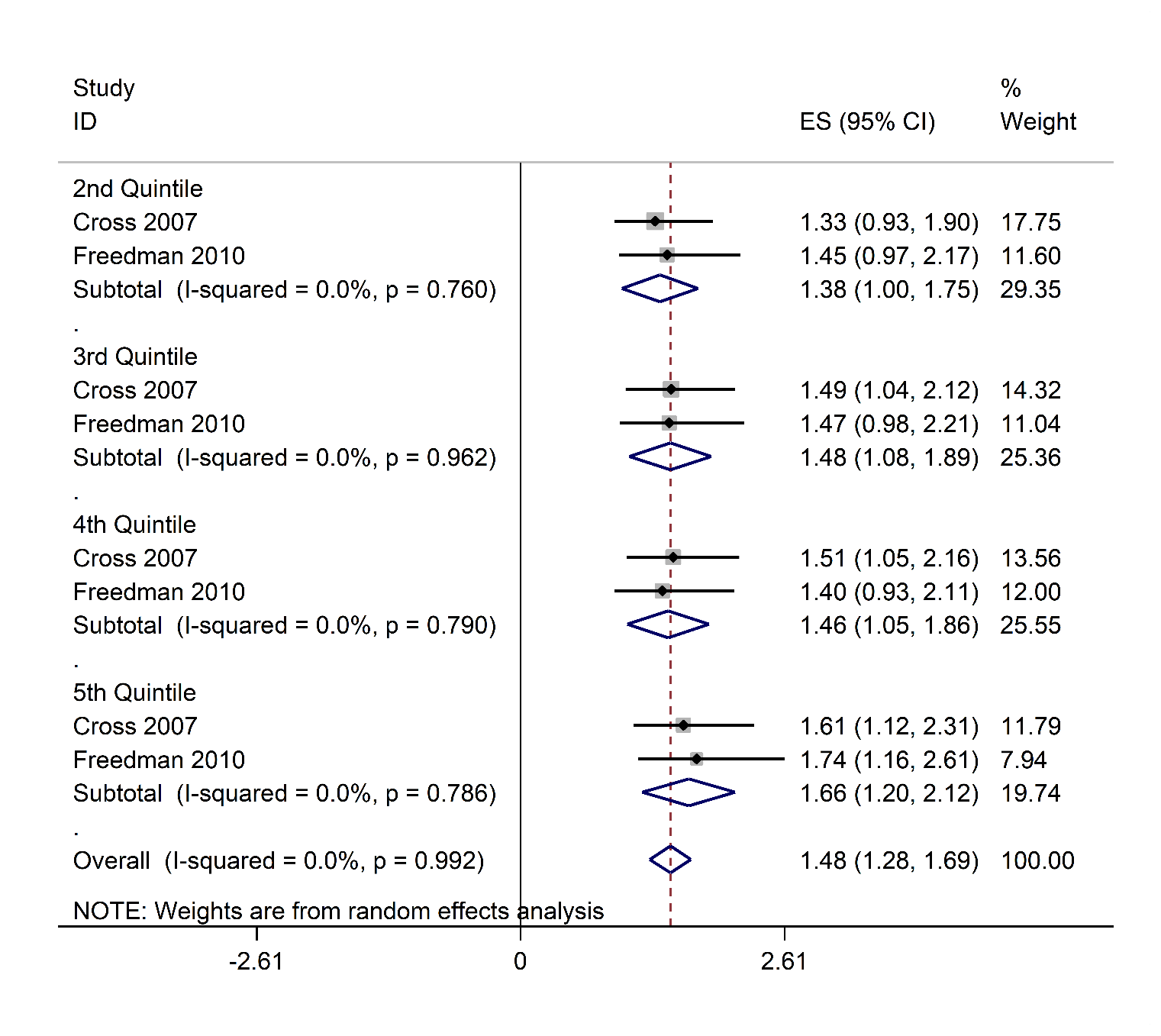
**

Forrest plot of the association between **liver** cancer and consumption of **red meat** classified as tertiles, quartiles, and quintiles


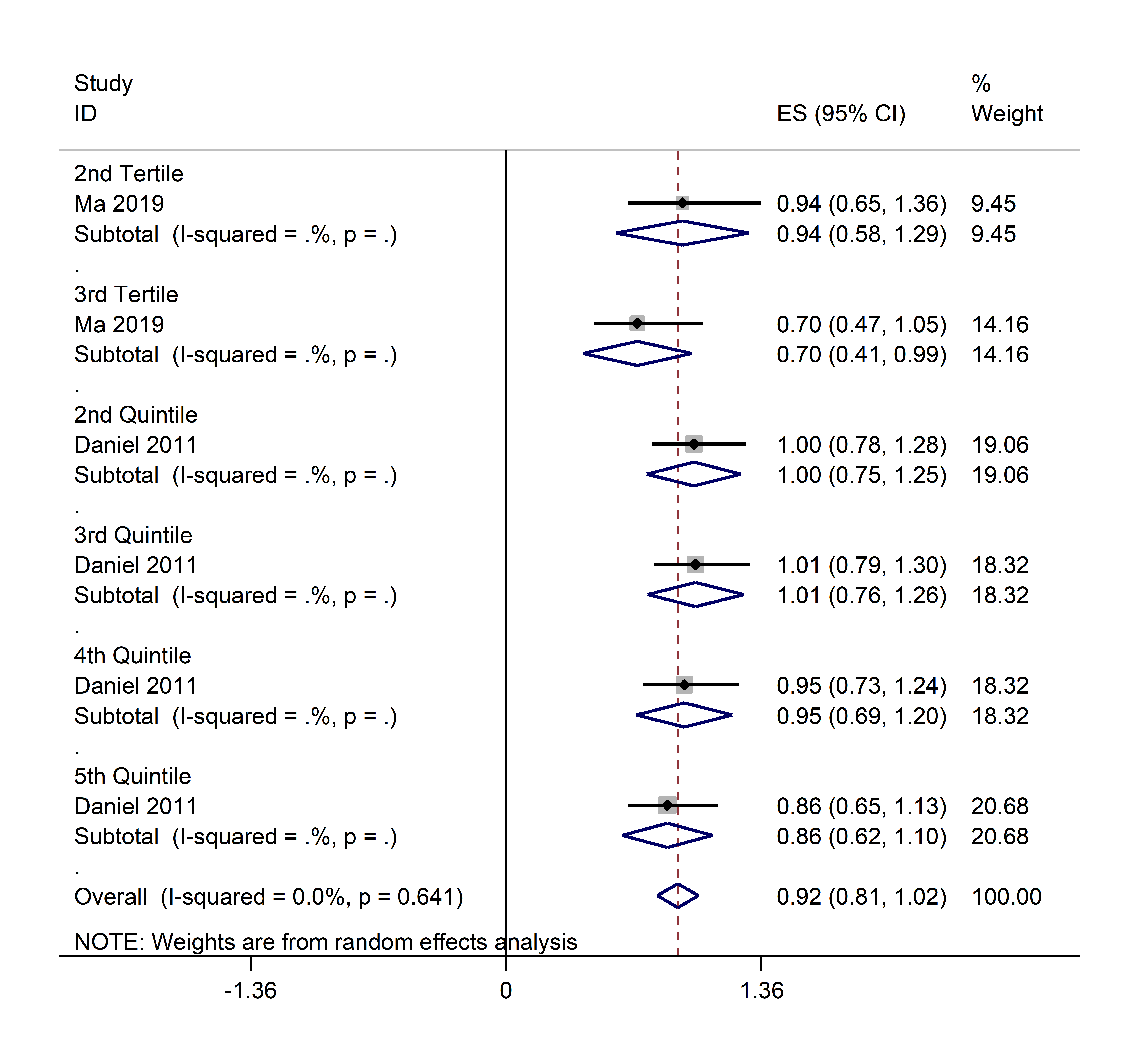


Forrest plot of the association between **liver** cancer and consumption of **fish** classified as tertiles, quartiles, and quintiles


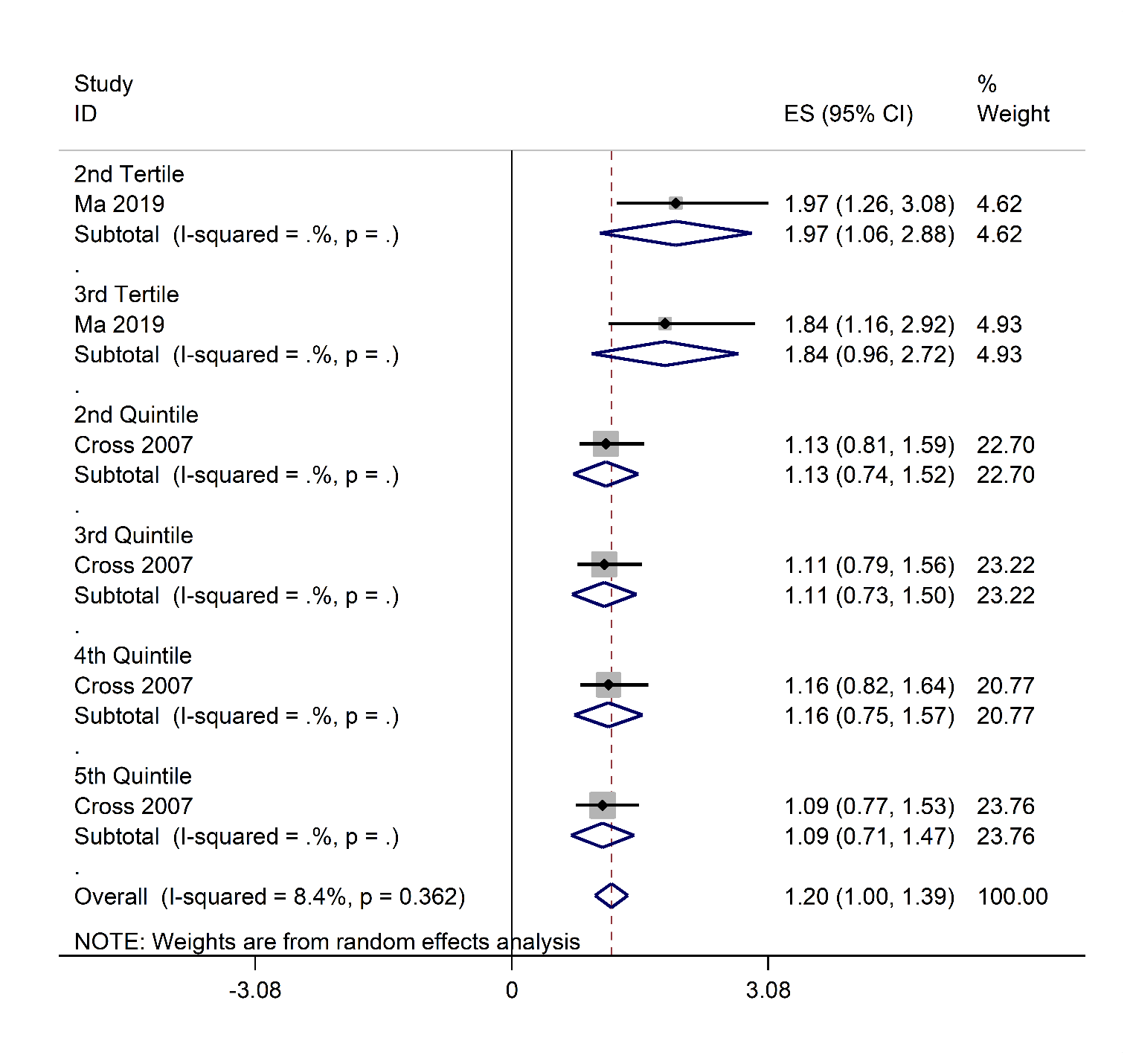


Forrest plot of the association between **liver** cancer and consumption of **processed meat** classified as tertiles, quartiles, and quintiles


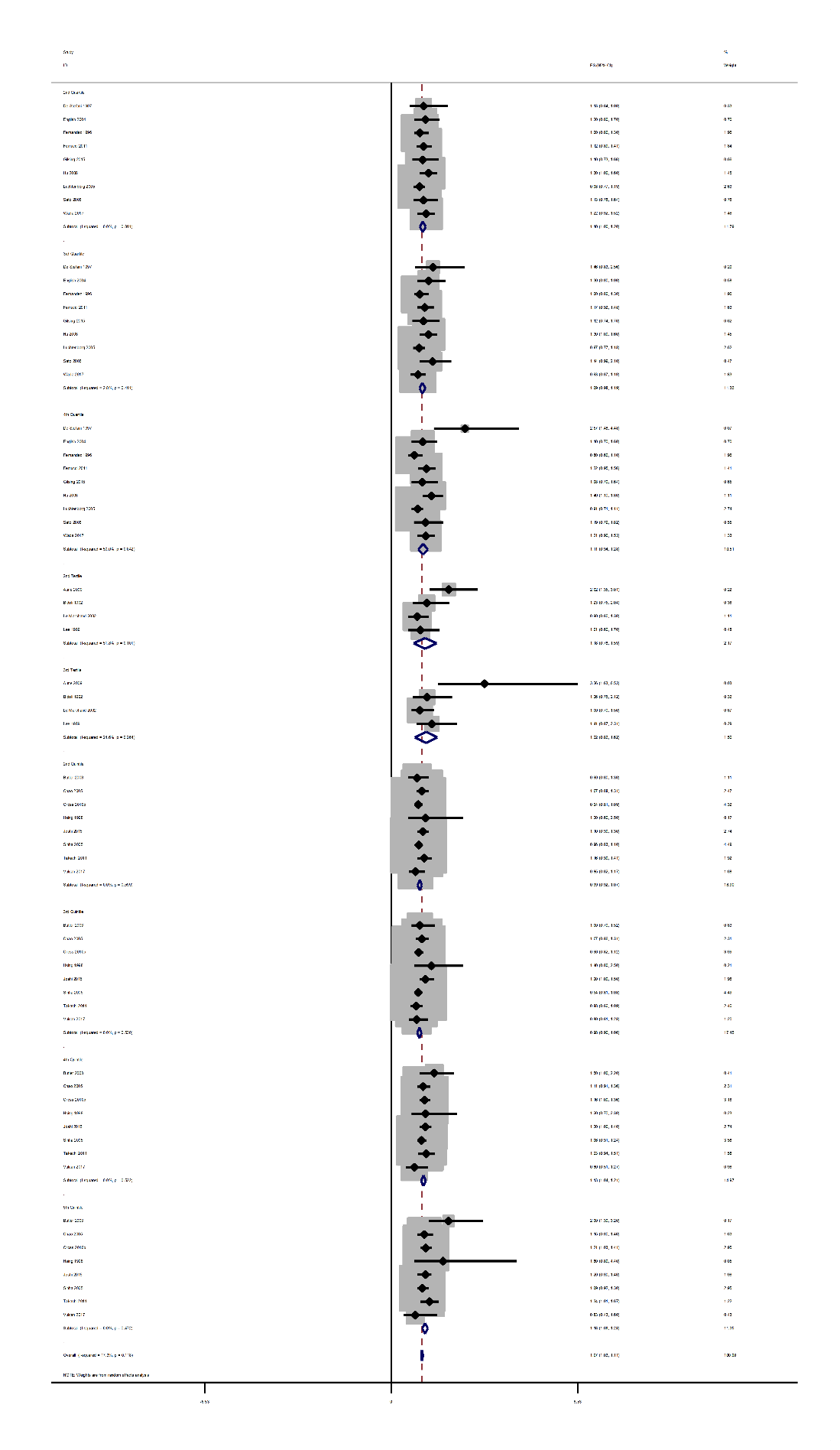


Forrest plot of the association between **colon** cancer and consumption of **red meat** classified as tertiles, quartiles, and quintiles

**
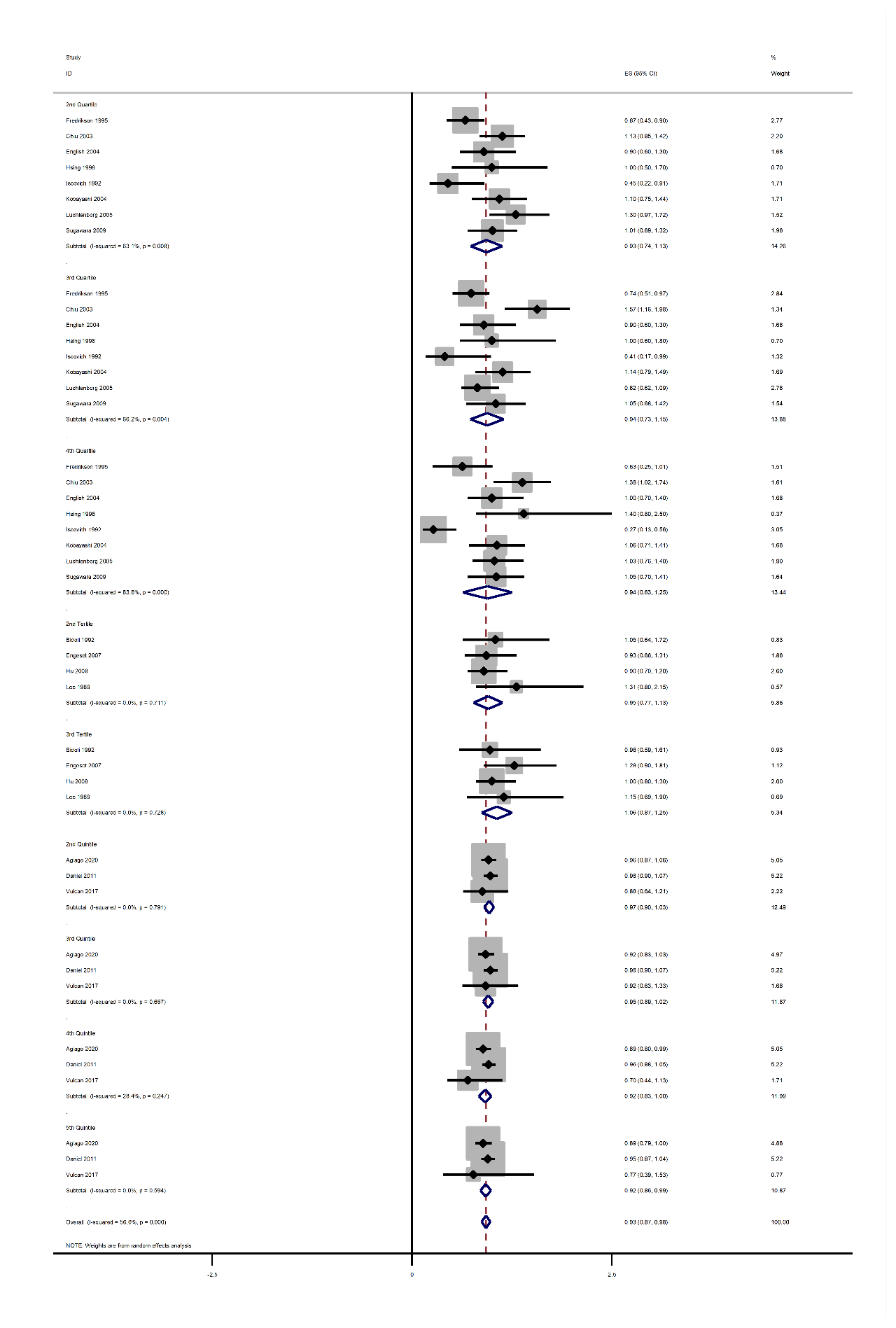
**

Forrest plot of the association between **colon** cancer and consumption of **fish** classified as tertiles, quartiles, and quintiles

**
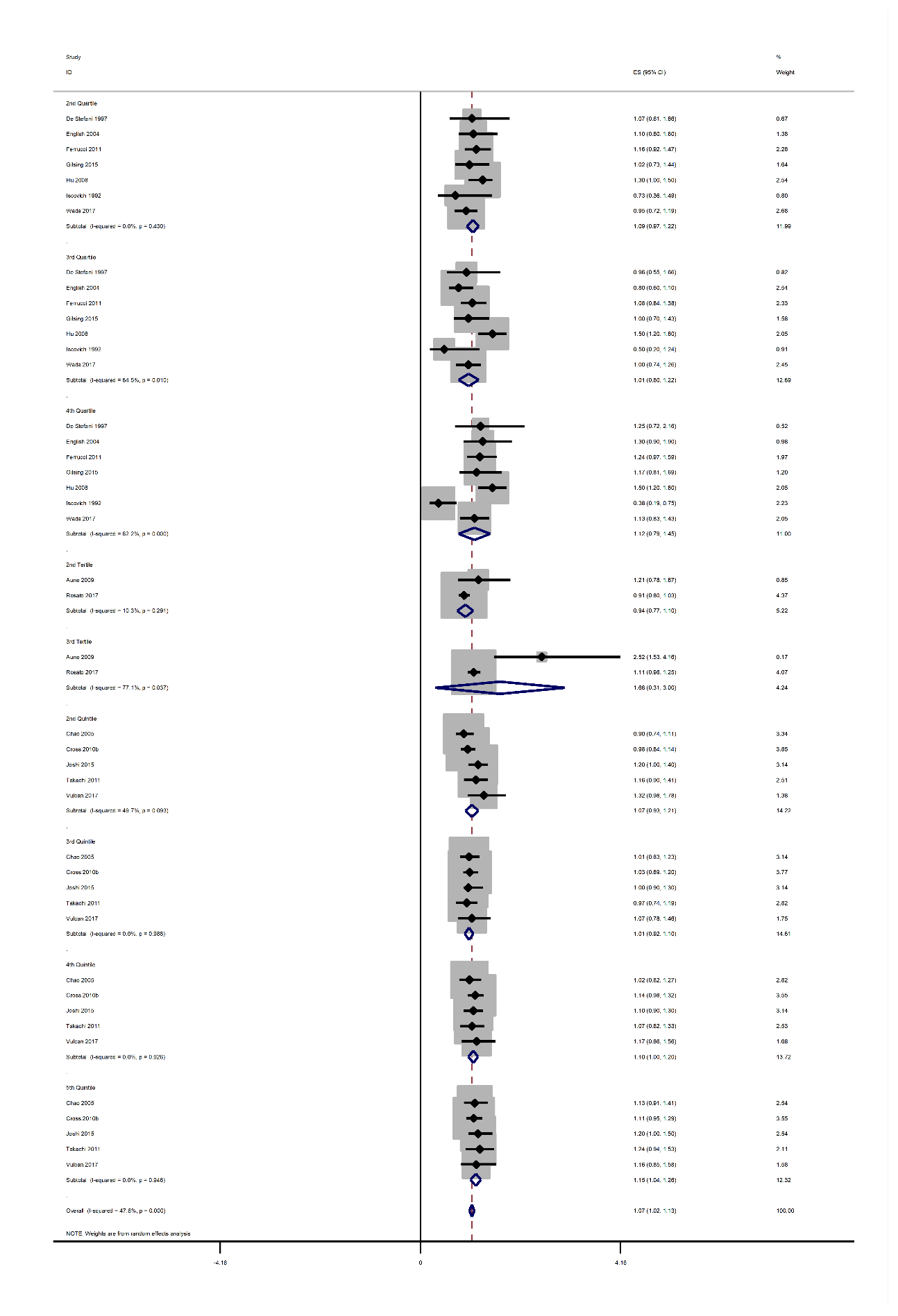
**

Forrest plot of the association between **colon** cancer and consumption of **processed meat** classified as tertiles, quartiles, and quintiles

**
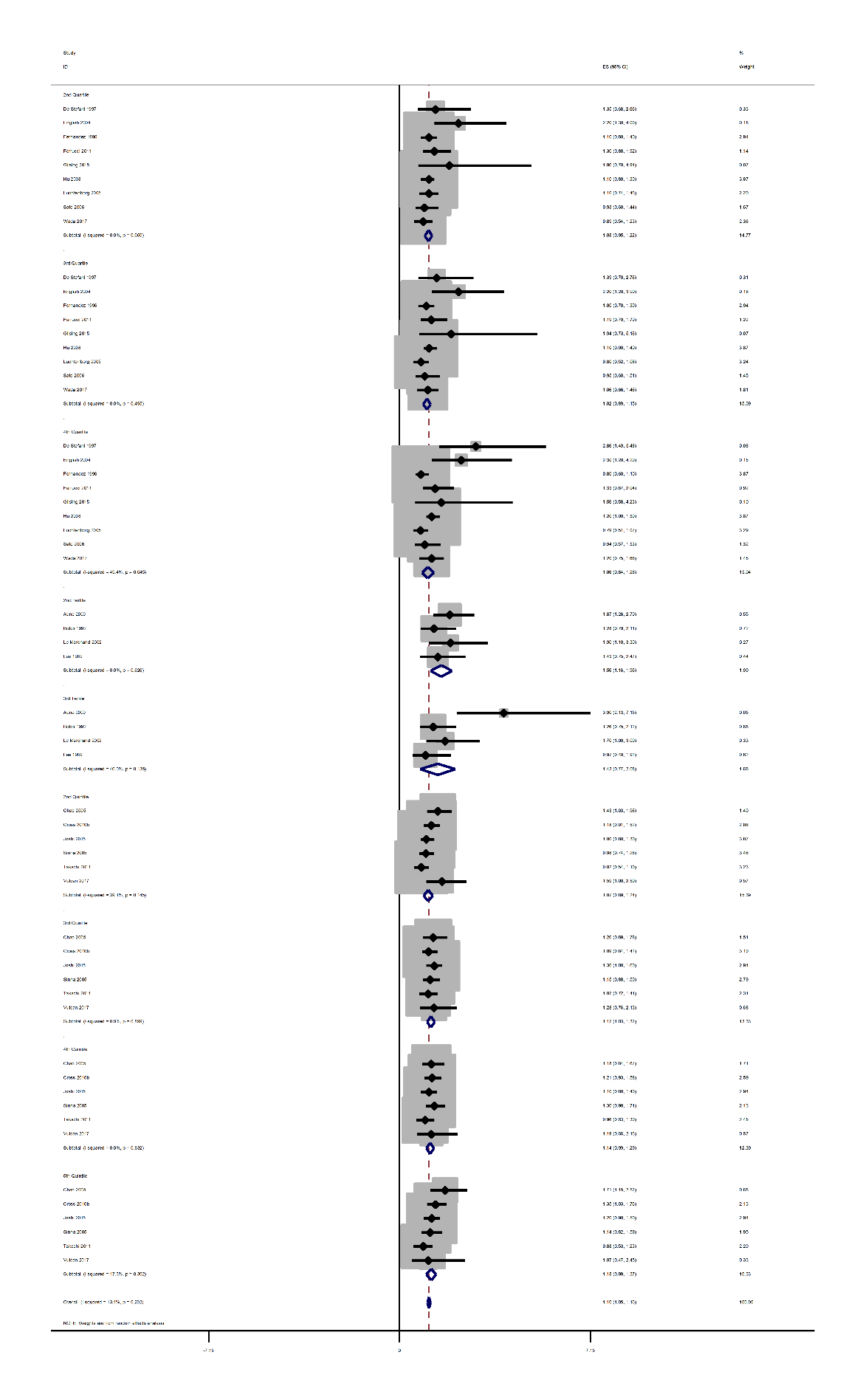
**

Forrest plot of the association between **rectal** cancer and consumption of **red meat** classified as tertiles, quartiles, and quintiles

**
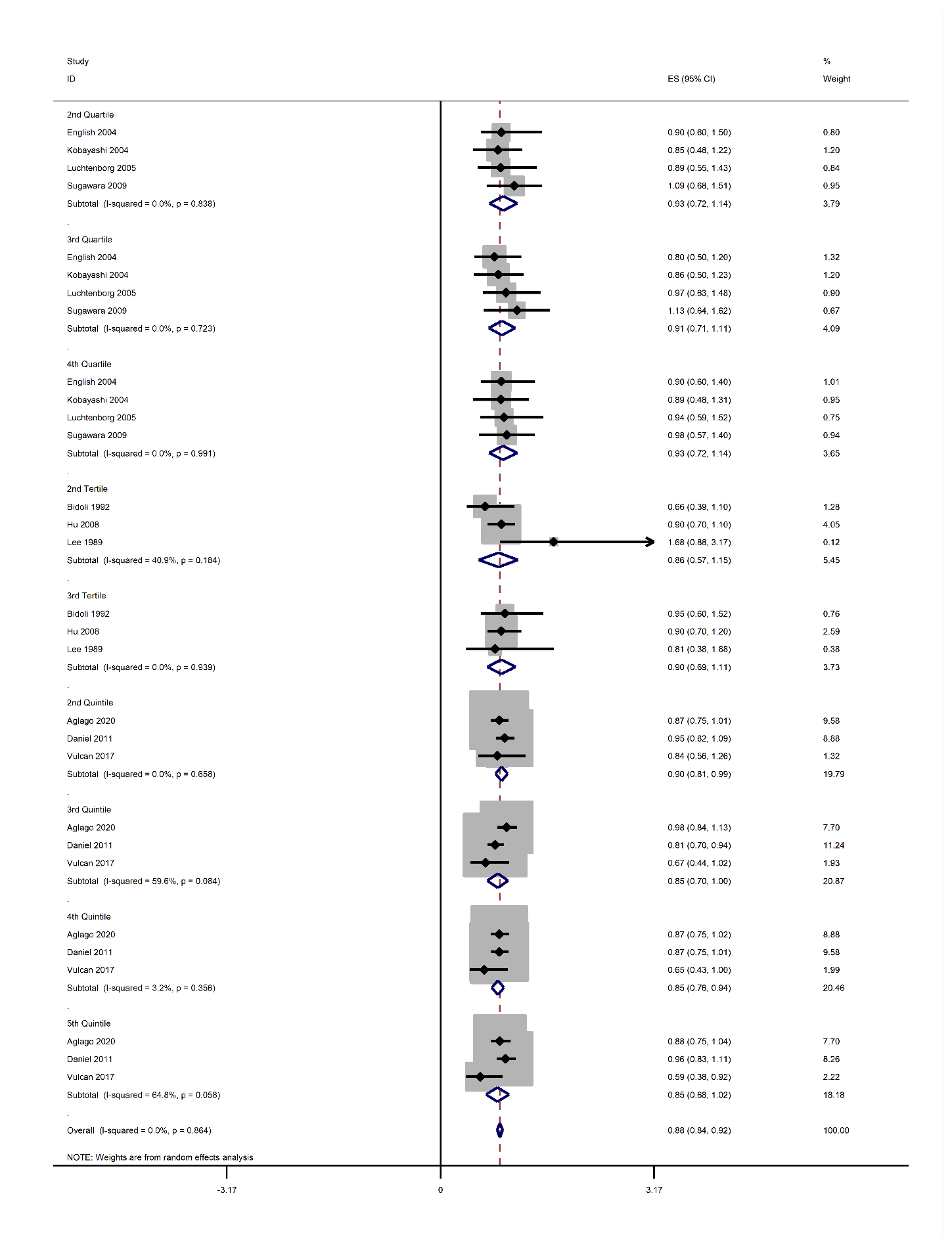
**

Forrest plot of the association between **rectal** cancer and consumption of **fish** classified as tertiles, quartiles, and quintiles

**
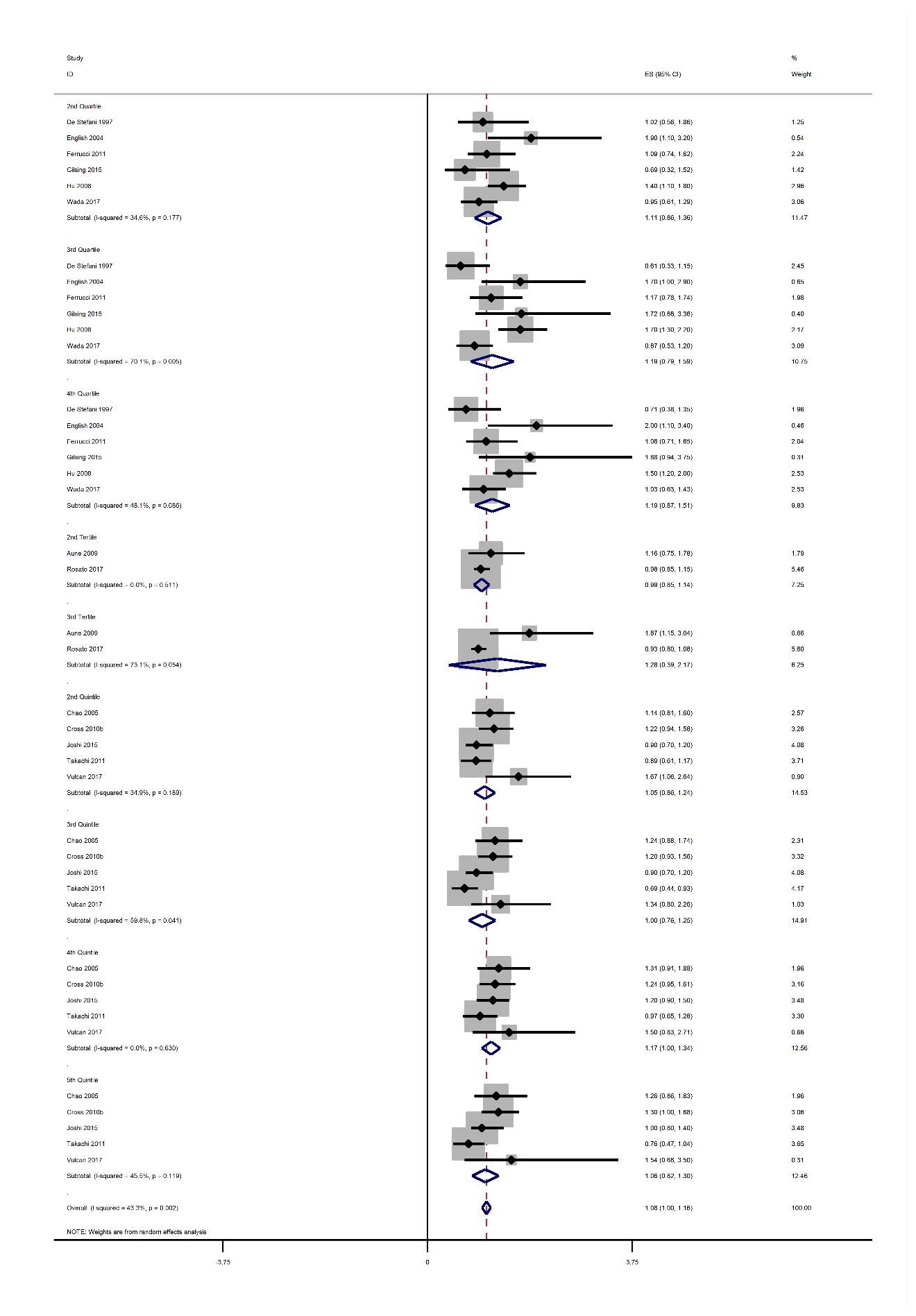
**

Forrest plot of the association between **rectal** cancer and consumption of **processed meat** classified as tertiles, quartiles, and quintiles

**
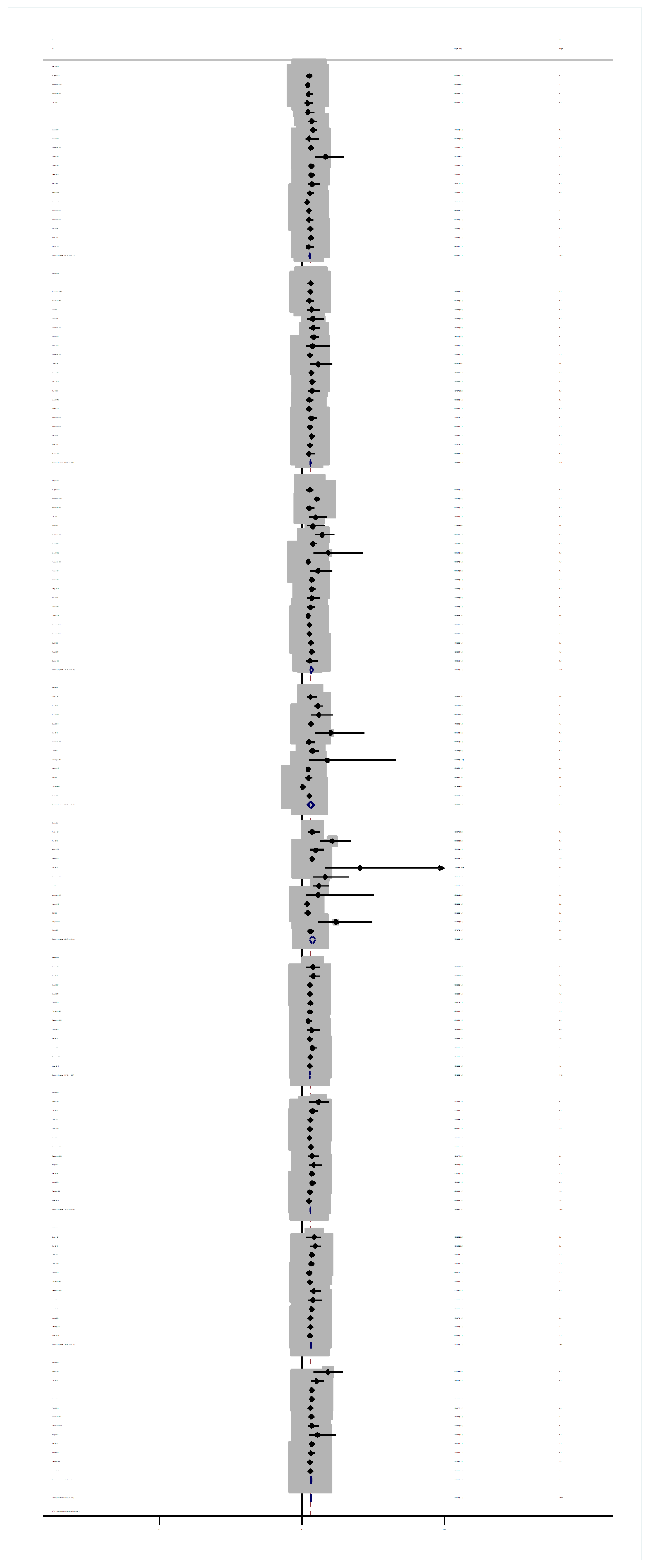
**

Forrest plot of the association between **colorectal** cancer and consumption of **red meat** classified as tertiles, quartiles, and quintiles

**
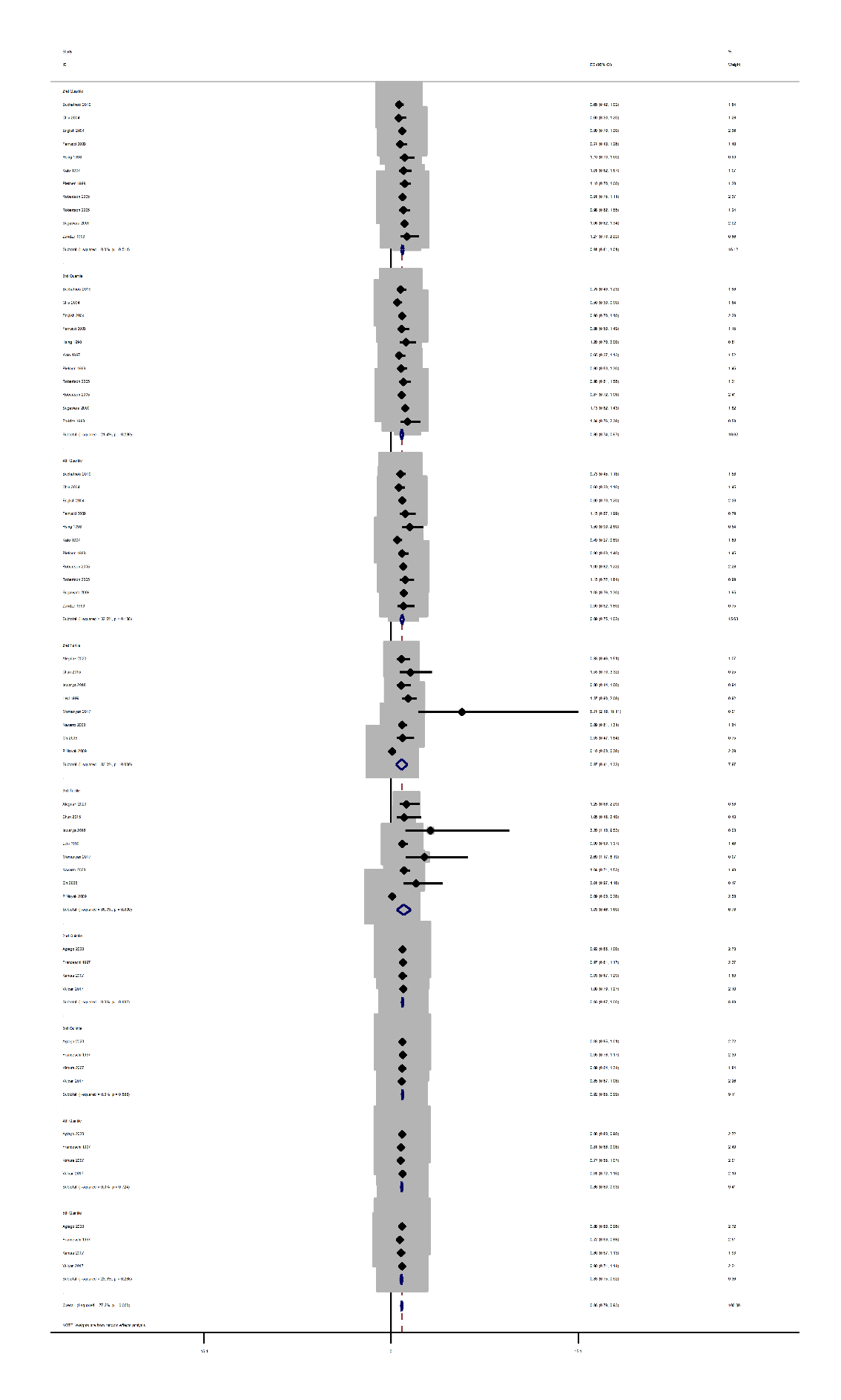
**

Forrest plot of the association between **colorectal** cancer and consumption of **fish** classified as tertiles, quartiles, and quintiles

**
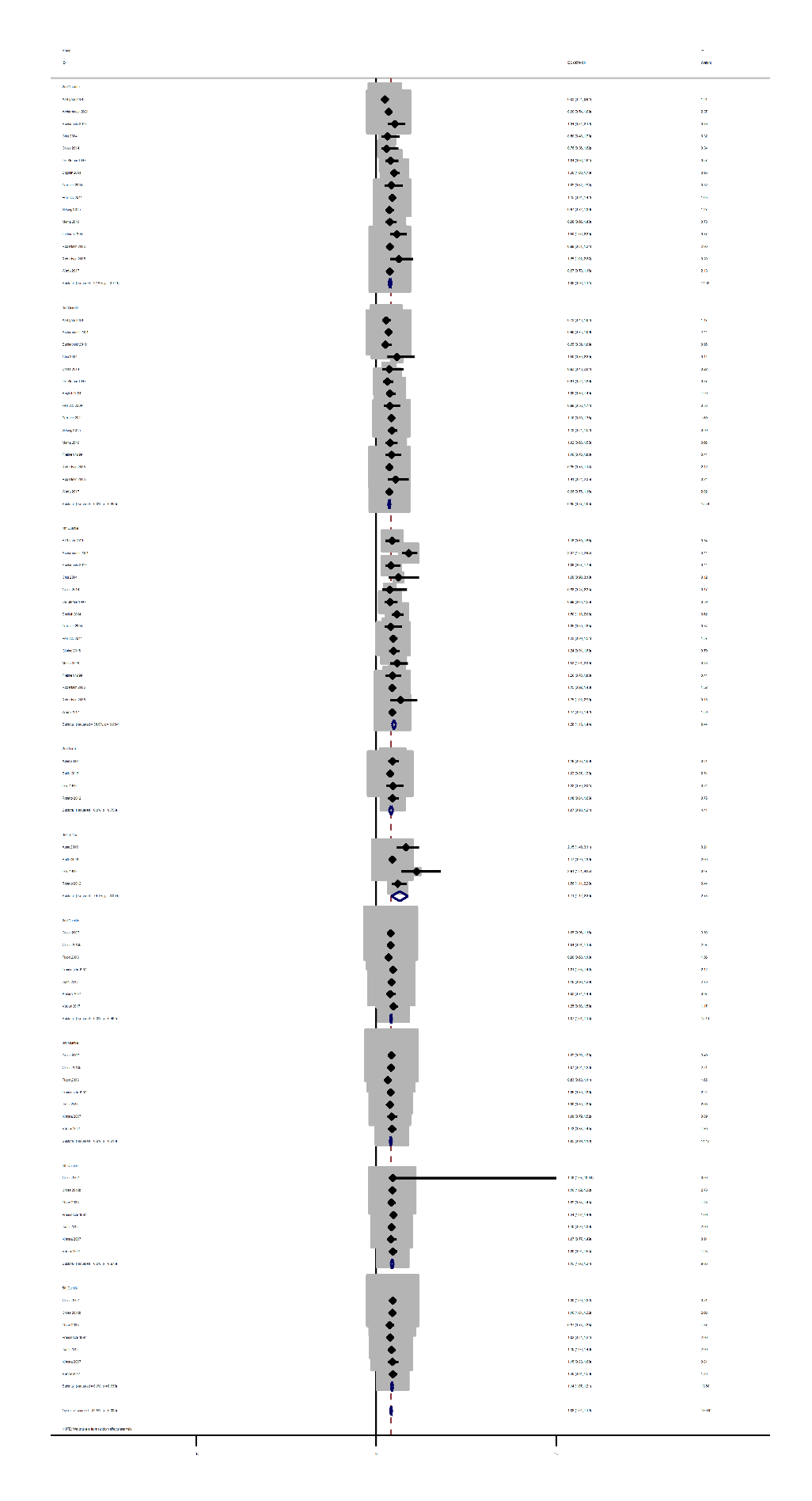
**

Forrest plot of the association between **colorectal** cancer and consumption of **processed meat** classified as tertiles, quartiles, and quintiles
